# Non-invasive tumor genotyping using radiogenomic biomarkers, a systematic review and oncology-wide pathway analysis

**DOI:** 10.18632/oncotarget.24893

**Published:** 2018-04-13

**Authors:** Robin W. Jansen, Paul van Amstel, Roland M. Martens, Irsan E. Kooi, Pieter Wesseling, Adrianus J. de Langen, Catharina W. Menke-Van der Houven van Oordt, Bernard H.E. Jansen, Annette C. Moll, Josephine C. Dorsman, Jonas A. Castelijns, Pim de Graaf, Marcus C. de Jong

**Affiliations:** ^1^ Department of Radiology and Nuclear Medicine, VU University Medical Center, Amsterdam, The Netherlands; ^2^ Department of Clinical Genetics, VU University Medical Center, Amsterdam, The Netherlands; ^3^ Department of Pathology, VU University Medical Center, Amsterdam, The Netherlands; ^4^ Department of Pathology, Princess Máxima Center for Pediatric Oncology and University Medical Center Utrecht, Utrecht, The Netherlands; ^5^ Department of Respiratory Diseases, VU University Medical Center, Amsterdam, The Netherlands; ^6^ Department of Medical Oncology, VU University Medical Center, Amsterdam, The Netherlands; ^7^ Department of Ophthalmology, VU University Medical Center, Amsterdam, The Netherlands

**Keywords:** radiogenomics, non-invasive, genotyping, biomarker, precision medicine

## Abstract

With targeted treatments playing an increasing role in oncology, the need arises for fast non-invasive genotyping in clinical practice. Radiogenomics is a rapidly evolving field of research aimed at identifying imaging biomarkers useful for non-invasive genotyping. Radiogenomic genotyping has the advantage that it can capture tumor heterogeneity, can be performed repeatedly for treatment monitoring, and can be performed in malignancies for which biopsy is not available. In this systematic review of 187 included articles, we compiled a database of radiogenomic associations and unraveled networks of imaging groups and gene pathways oncology-wide. Results indicated that ill-defined tumor margins and tumor heterogeneity can potentially be used as imaging biomarkers for 1p/19q codeletion in glioma, relevant for prognosis and disease profiling. In non-small cell lung cancer, FDG-PET uptake and CT-ground-glass-opacity features were associated with treatment-informing traits including *EGFR*-mutations and *ALK*-rearrangements. Oncology-wide gene pathway analysis revealed an association between contrast enhancement (imaging) and the targetable VEGF-signalling pathway. Although the need of independent validation remains a concern, radiogenomic biomarkers showed potential for prognosis prediction and targeted treatment selection. Quantitative imaging enhanced the potential of multiparametric radiogenomic models. A wealth of data has been compiled for guiding future research towards robust non-invasive genomic profiling.

## INTRODUCTION

**Figure 1:**
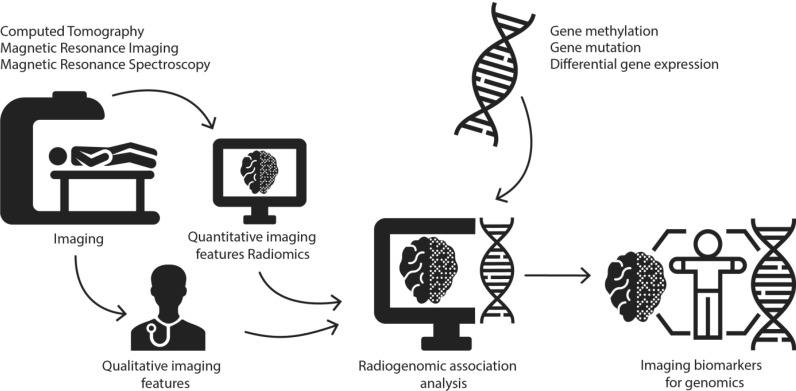
Illustration of the research methods of radiogenomics

Considerable progress had been made in developing targeted therapies for genomic subtypes in cancer, but patient selection for these therapies can be challenging. Radiogenomics (sometimes imaging genomics) is a new, rapidly evolving field of research aimed at developing tools for non-invasive genotyping by identifying imaging biomarkers for genomic subtypes [1–3]. Radiogenomic analysis refers to the integration of radiophenotypes and genomic data in order to find radiogenomic associations (Figure 1). Radiogenomic analysis can be performed using qualitative- or quantitative (computer-extracted, radiomics) imaging features, which can be used as individual biomarkers or can be incorporated in multiparametric prediction models.

Radiogenomics yields considerable advantages for genotyping. Firstly, tumor genetic heterogeneity can be captured using radiogenomics. Biopsy-based genotyping in the clinical setting is generally confined to a single sample, although multiregional genotyping has been performed effectively to capture tumor heterogeneity [4–6]. Radiogenomic biomarkers have shown a great potential for capturing tumor heterogeneity non-invasively [7, 8]. Secondly, a non-invasive method can be performed repeatedly, and is therefore eminently suitable for treatment follow-up. In addition, radiogenomic markers are important for tumors for which biopsy is unavailable (e.g. glioma, retinoblastoma) [9]. Finally, radiogenomics is fast and cost-effective, generally using routine clinical imaging. Several non-systematic reviews were published on radiogenomics [2, 3, 10–19]. The main purpose of this systematic review was to provide a comprehensive oncology-wide database of radiogenomic associations, and to review their clinical usefulness. A secondary objective was to assess radiogenomics on a pathway-level instead of a gene-level; to perform oncology-wide gene pathway analysis in order to identify relations between imaging and oncopathways.

## RESULTS

### Database of imaging-genomics associations

**Figure 2:**
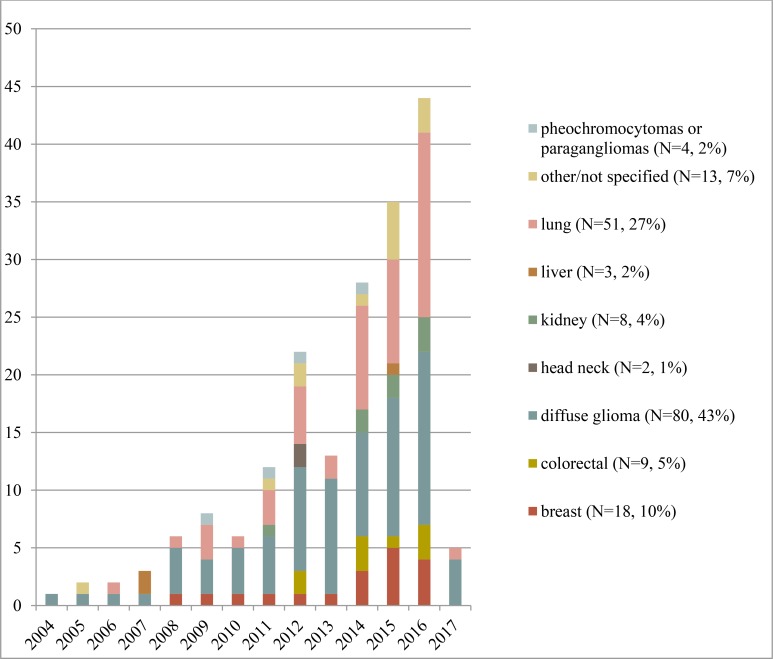
The number of included articles per type of neoplasm, by year of publication

We included 187 articles published between July 2004 and February 2017. A PRISMA flow diagram for the inclusion process is available in the Supplementary Table 1. Figure 2 illustrates the exponential growth of publications on a year-over-year basis. The major groups reflected diffuse glioma (*n* = 79, 42%), non-small cell lung cancer (NSCLC) (*n* = 51, 27%), and breast cancer (*n* = 18, 10%). Often, studies used multiple modalities; 105 studies used MRI (56%), 80 CT (43%), 44 FDG-PET (24%), and 5 mammography (3%). In 59/187(32%) articles biological clarifications for imaging-genomics relations were identified. The 2440 identified radiogenomic associations in the database are presented as a pivot table, which provides an easy graphical interface to perform data queries using Microsoft Excel (2010/2013) (Supplementary Table 2). Study characteristics and quality assessment are available in the Supplementary Table 3. The results section focuses on repeatedly identified imaging-genomics associations with possible clinical application.

### Glioma: *IDH*-mutation status and 1p/19q codeletion

The 2016 World Health Organisation (WHO) Central Nervous System (CNS) tumor classification uses a combined phenotypic (histology) and genotypic classification for diffuse glioma (diffuse astrocytic and oligondendroglial tumors grade II-IV) [20]. The major genetic traits are *IDH* mutation and 1p/19q codeletion, both associated with a more favourable prognosis [21–26]. Table 1 summarizes radiogenomics for *IDH*-status in glioblastoma (GBM, grade IV glioma), while Table 2 and Supplementary Table 4 show radiogenomics results for *IDH*-status and 1p/19q codeletion in grade II-III glioma. On MRI, *IDH*-mutated cases were characterised by increased perfusion parameters in both glioblastoma (higher tumor blood flow) [27] and grade II glioma (higher relative cerebral blood volume) [28]. Additionally, detection of 2-hydroxyglutarate (2-HG) with MR-spectroscopy (MRS) was a strong predictor of *IDH*-mutations in glioblastoma (grade IV) [29], grade II-III glioma [30] and grade II-III-IV glioma [31]. Furthermore, four studies applied multiparametric models predicting *IDH*-status based on qualitative and quantitative features (Supplementary Table 5) [32–35]. 1p/19q codeletion, a pathognomonic trait that defines a distinct glioma entity (oligodendroglioma), was associated with ill-defined tumor borders [36–39] and tumor heterogeneity [36–39] on MRI of grade II-III glioma. Results of one study revealed combined perfusion and MRS metabolite ratios can discriminate tumors with 1p/19q loss of heterozygosity with an accuracy of 72% [40]. MGMT-methylations status is relevant for glioblastoma, as it could aid patient selection for adjuvant temozolamide chemotherapy [41–43]. Supplementary Table 6 summarizes findings of studies assessing *MGMT*-methylation status [27, 32, 44–52] in glioblastoma. *MGMT*-methylated tumors showed higher apparent diffusion coefficient (ADC) values on diffusion weighted-MRI (DW-MRI) in four out of five studies [27, 44–47]. Supplementary Table 7 summarizes the findings of studies correlating MR features with *EGFR*-status in diffuse glioma. MR perfusion parameters correlated with *EGFR*-amplification [53], and *EGFR*-mutation status in glioblastoma [54, 55] and with *EGFR*-expression in grade II-II oligodendroglioma [56] and grade III-IV glioma [57].

**Table 1:** Overview of radiogenomics for predicting IDH mutation status in glioblastoma (grade IV), *p*-values for associations

					Necrosis	Enhancement		Diffusion	Perfusion	MRS	Other	
*IDH*-mutations	*Glioma grade*	*Author*	*Year of pub.*	*N*	MR Necrosis	MR CE Contrast enhancement	MR CE Contrast enhancement pattern	MR ADC apparent diffusion coefficient (mean; min)	MR TBF tumour blood flow (mean absolute; relative)	MRS (magnetic resonance spectroscopy) 2-HG metabolite imaging	MR Edema (brain; peritumoural)	MR Nonenhanced tumour
	Grade IV	Choi [161]	**2012**	29						**<0.001**		
		Gutman [192]	**2013**	75	0.19	0.08					0.6	0.23
		Wang [200]	**2015**	280		**<0.001** **0.003**	0.621				0.395	
		Yamashita [27]	**2016**	55	**<0.05**			>0.05 >0.05	**<0.001** **<0.001**			

**Table 2:** Overview of radiogenomics for predicting IDH mutation status in glioma grade II-III, *p*-values for associations

					Volume	Margin	Location	Calcification	Heterogeneity	Enhancement	Perfusion	MRS		PET
*IDH*-mutations	*Glioma grade*	*Author*	*Year of pub.*	*N*	**MR Tumour volume**	**MR Tumour margin well poorly defined**	**MR Location features**	**CT Calcifications**	**MR Heterogeneity**	**MR CE contrast enhancement tumour**	**MR CBV cerebral blood volume 90th percentile**	**MRS (magnetic resonance spectroscopy) 2-HG metabolite imaging**	MRS Magnetic resonance spectroscopy other metabolites	PET FDG SUV max ratio
	Grade II	Yu [201]	**2017**	92			**0.007**							
		Kickingreder [28]	**2015**	73							AUC 0.922 OR 031 **p 0.01**			
		Wang [202]	**2015**	146			**<0.05**							
		Metellus [203]	**2010**	47	0.047	**0.007**	**0.004**		0.82	0.99				
	Grade II, III	Metellus [204]	**2011**	33										0.775
		Pope [30]	**2012**	24								**0.003**		
		Saito [205]	**2016**	250				**0.0004**						
	Grade II, III, IV	Nakae [206]	**2016**	167									**<0.001**–0.49	
		Kalinina [31]	**2012**	75								Sens 0.960 Spec 0.952 ***P* < 0.001**		

### Multiparametric modelling for radiogenomics in diffuse glioma

Supplementary Table 5 summarizes findings of studies incorporating quantitative imaging and genomics data in multiparametric models. Seven studies created prognostic models using whole-genome data and imaging [58–65]. Four studies successfully correlated quantitative perfusion traits with angiogenic gene signatures [66–69].

### Non-small cell lung carcinoma: *EGFR*-mutations, *ALK*-rearrangements, *KRAS*-mutations

Radiogenomic studies for NSCLC follow the emerging field of personalized, genotype-directed therapy for NSCLC (Supplementary Table 8). However, divergent findings were reported on the association between presence of *EGFR*-mutation (treatable with tyrosine kinase inhibitors, TKI) and imaging (standardized uptake value [SUV] on FDG-PET and proportion ground glass opacity [GGO] on CT). FDG-PET uptake was both negatively [70–77] and positively [78, 79] correlated with EGFR-mutations, while other studies found no correlation [74, 80–83]. Additionally, *EGFR*-mutated tumors were on average more solid (less GGO) [80, 84, 85], although not completely solid (some degree of GGO) [86–88]. However, one study showed an inverse relation [83] and other studies found no association [70, 78, 89–95]. Similarly, *ALK*-rearranged tumors (treatable with *ALK*-inhibitors) were more solid in two [95, 96] out of four [80, 85, 95, 96] studies compared with ALK-wild-type (wt) tumors. When using GGO to discriminate *ALK*-rearrangements from *EGFR*-mutations, *ALK*-rearranged tumors were more solid [80, 95]. Compared with *ALK*-wt, *ALK*-rearranged tumors had more spiculated [80] and lobulated [95] tumor margins on CT [80, 95]. Another driver mutation is *KRAS,* which is the most frequent driver mutation, but no CT or PET features were repeatedly associated with *KRAS*-mutation status (Supplementary Table 9).

### Quantitative imaging and multiparametric modeling for radiogenomics in lung cancer

Recent studies performed multiparametric modelling and quantitative imaging in NSCLC [97–104]. Individual quantitative texture features successfully identified *EGFR*-mutation (multiple comparisons) [103, 104], as did multiparametric models adopting quantitative (AUC = 0.74–0.91), qualitative, (AUC = 0.89) [87], or combined quantitative-clinical features (AUC = 0.70) [98]. Quantitative CT- and PET-features could also predict *ALK* or *ROS1/RET* fusions (sens = 0.73, spec = 0.70) [105]. For development of prognostic imaging biomarkers, two groups used quantitative imaging for predicting prognosis-related gene clusters and found a lower kurtosis value linked with poorer survival [99]. Additionally, a module of tumor size, edge shape, and sharpness could predict survival [97]. Similarly, the prognostic value of PET-imaging was explained from a genomic perspective using radiogenomic analysis [100, 101].

### Breast cancer

This review only included studies with analyses on a genomic level; imaging-receptor associations based on immunohistochemistry analysis were reviewed elsewhere [10]. High FDG-PET uptake was found for gene expression signatures for basal like, while low uptake was found for luminal like cases [106]. Low FDG-PET uptake was also associated with expression of oestrogen-receptor related genes [107]. Other studies associated luminal B genes with quantitative dynamic MRI-perfusion [108] and *BRCA*-mutations with sharp margins and rim enhancement on MRI [109], but these findings were not independently validated.

### Multiparametric modeling for radiogenomics in breast cancer

Radiogenomic imaging models were used in breast cancer in twelve studies [110–121]. Five studies [110, 112, 118, 120, 121] focussed on Oncotype Dx gene-expression score, which predicts recurrence in early-stage ER+/HER2- invasive cancers [122]. One [118] additionally assessed prognostic gene assays MammaPrint [123] and PAM50 [124, 125]. Enhancement heterogeneity, on either quantitative perfusion [110, 120] or quantitative texture analysis [118], was associated with high-risk assays in three studies. Rapid contrast uptake predicted high-risk Oncotype Dx in two studies [110, 112]. To increase insight into underlying genomics of MR-perfusion parameters a study associated gene expression profiles with a heterogeneous centripetal perfusion phenotype [116] and another study associated perfusion parameters with regulatory non-coding transcripts of RNA associated with early metastasis [126]. Radiogenomics was applied for monitoring of anti-VEGF treatment by measuring pre- and post-treatment perfusion and associated differential gene expression [119]. Additionally, a qualitative imaging model including tumor heterogeneity and enhancement predicted expression of immune-response genes [114]. Combined analysis of tumor shape (lobulated oval) on mammography and MRI showed significant correlations with Oncotype Dx [121].

### Colorectal cancer

Nine studies were included for CRC [127–135]. Higher FDG-PET uptake was found for *KRAS*-mutated tumors in five [128, 129, 133, 136, 137] out of six [134] studies. Additionally, *KRAS*-mutations could be predicted in a multiparametric model using high FDG-PET uptake in addition to CT texture and perfusion features [137]. Cases that were both *KRAS*- and *TP53*-mutated also showed a higher SUVmax [129], as did cases that belonged to the group “*KRAS*- or *BRAF*–mutated” [132].

### Renal cell carcinoma

Eight studies were included for RCC [138–145]. *BAP1*-mutations, associated with invasive disease [146, 147], showed more calcifications (CT) in two studies, although in one non-significantly (*p* = 0.09 [138]; *p <* 0.001 [140]). A radiogenomic risk score, based on a multiparametric qualitative CT model successfully predicted a predefined prognostic gene signature in RCC [142, 143]. One study identified genetic underpinnings of an imaging-based complication prediction score (PADUA) [145].

### Hepatocellular carcinoma

Three studies were included for HCC [148–150]. Tumors with ill-defined margins on CT showed high expression of a gene expression signature for doxorubicin-sensitivity [150]. Additionally, targetable high *VEGF*-expression [151] was related to attenuation, heterogeneity and tumor margins on CT [148]. A gene signature of microvenous invasion (indicating poor prognosis) can be predicted by a CT biomarker including presence of small intratumoral internal arteries and the absence of hypodense halos [148]. A different genomic score for venous invasion was correlated with CT intratumoral arteries and margins [150].

### Radiogenomics in other malignancies

In paraganglioma, four studies found higher PET SUV-values for *SDHx*-mutated tumors [152–154] or *SHDx* and *VHL*-mutated tumors [155], relevant for heritability risk assessment. In head neck tumors, *EGFR*-expression was related to CT invasion, mass effect, size/volume [156] and lower capillary permeability on perfusion CT [157]. Additionally, 14 studies each reported on radiogenomic associations of 14 other malignancies, respectively (Table 3).

**Table 3:** Radiogenomics in other malignancies

Diagnosis	Study	Year	*N*	Study design (radiogenomic analysis)	Genetic feature	Significantly correlated imaging feature	*p*-value
Cervical cancer	Halle [194]	2012	187	Prediction of expression of a set of hypoxia-induced genes with a DCE-MRI imaging model	Hypoxia-induced genes set (31)	DCE-MRI imaging feature model (Abrix)	Multiple significant finding, appendix S2
Diffuse large B-cell lymphoma	Lanic [232]	2011	57	Multiparametric modelling incorporating imaging (PET) and genomics to predict prognosis	Germinal center B cell-like (GCB) vs Activated B cell-Like (ABC) (gene set expression)	PET High SUV-uptake	0.0291
Extraskeletal myxoid chondrosarcoma	Tateishi [233]	2005	19	Describing MR findings in 19 extraskelatal myxoid chondrosarcoma patients	EWS-CHN translocation vs other cytogenic variants	MR Peripheral enhancement	<0.05
Lipoma and atypical lipomatous tumor/ well-differentiated liposarcoma	Brisson [234]	2012	87	Identification of CT imaging biomarkers for *MDM2* amplifications (classified as atypical lipomatous tumor/well-differentiated liposarcoma)	*MDM2* amplification	CT Lesion size >10 cm	0.011
						CT Location: lower limb CT Solid fat content	0.007
							0.002
Melanoma brain metastases	Bordia [235]	2016	98	Identification of MR imaging features of melanoma brain metastasis associated with genetic profiles and survival	*BRAF* mutation	MR Size of lesions	<0.05
						MR Edema MR Hyperintensity T1 MR Hyperintensity T2 compared to grey matter MR Enhancement MR Diffusion characteristics	<0.05
							<0.05
							<0.05
							<0.05
							<0.05
Multiple myeloma	Mai [236]	2016	164	Identification of genetic underpinnings of qualitative MR imaging patterns	Any adverse cytogenetics (chrom. 17p deletion/t(4;14)/chrom. 1q21 gain)	MR Diffuse patterns	0.02
							0.04
Ovarian cancer (high grade serous)	Vargas [237]	2015	46	Qualitative and quantitative assessment of CT features to predict gene expression subtypes (Clovar)	Mesenchymal gene expression subtype (Clovar)	CT Mesenteric infiltration	0.002–0.005
						CT Diffuse peritoneal involvement	0.004–0.012
Neuroblastoma	Liu [238]	2015	42	Use of FDG-PET and FDOPA-PET for distinguishing neuroblastoma genomic subtypes	*DDC* expression	PET FDG ratio to FDOPA negative	0.02
					*HK2* expvression	PET FDG ratio to FDOPA positive	<0.0001
					*Mycn* amplification	PET FDG ratio to FDOPA positive	0.002
					*SLC6A2* expression	PET FDOPA uptake	0.004
Medulloblastoma	Perreault [239]	2014	47	Qualitative assessment of MR imaging features to predict 4 molecular subgroups (wingless, sonic hedgehog, group 3, and group 4)	Group 3/4	MR Tumor location within the midline fourth ventricle	<0.001
					Wingless	MR Tumor location cerebellar peduncle/cerebellopontine angle cistern	<0.001
					Sonic hedgehog	MR Tumor location cerebellar hemispheres	<0.001
					Group 4	MR No/minimal contrast enhancement	<0.001
					Group 3	MR Ill-defined tumor margins	0.03
Pilocytic astrocytoma	Zakrzewski	2015	86	Identification of transcriptional profiles related to radiological findings	Transcriptional profiles	MR: Solid or mainly solid, Cystic/Enhanced, Cystic/Non enhanced, Largely necrotic	No relation found
Pancreatic cancer	Shi [131]	2015	60	Correlation of PET-imaging features with major oncogenomic alterations	*CDKN2A* loss of heterozygosity	PET (MTV and TLG)	0.029 0.021 resp.
					*SMAD4* loss of heterozygosity	PET (MTV and TLG)	0.001 0.001 resp.
					*TP53* mutation	PET (MTV and TLG)	0.001 0.001 resp.
Prostate cancer	Stoyanova [240]	2016	6	Multiparametric quantitative imaging association with whole genome(gene ontology) and predefined genomic classifiers	Whole genome expression, predefined genomic classifiers	Multiple quantitative imaging features including DCE-MRI	Significant findings for both predefined gene classifiers as newly identified pathways
Thyroid cancer	Nagarajah [241]	2015	81	Identification of PET-imaging features related to BRAFv600E mutation	BRAFv600E mutation	PET SUVmax	0.019

### Oncology-wide comparison of radiogenomic correlations and gene pathway analysis

**Figure 3:**
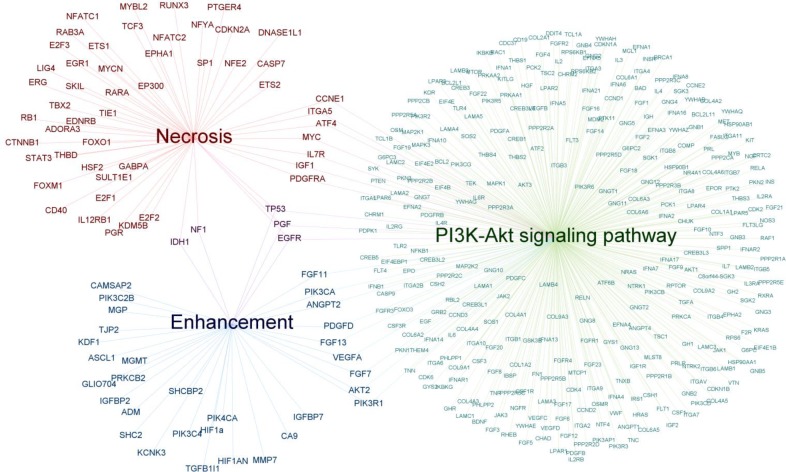
Genetic traits associated with either enhancement or necrosis Genes associated with degree of enhancement (*N* = 37) and genes associated with necrosis (*N* = 55) are depicted. The genes *IDH1*, *NF1*, *TP53*, *PGF* and *EGFR* are shared between both groups. The two gene sets were both enriched for PI3K-Akt signalling (enhancement: 12 common genes, Bonferroni corrected *p* < 0.0001; necrosis: 10 common genes, Bonferroni corrected *p* = 0.0005).

Looking at the molecular pathway-level, gene ontology analysis reveals associations between imaging groups and gene pathways in cancer (KEGG) oncology-wide (Table 4). Distinct cancer pathways were associated with imaging group of necrosis (55 genes/6 pathways) and of contrast enhancement (37 genes/6 pathways). Enhancement features (degree) were associated with the targetable signalling pathways of VEGF (*p* < 0.0001) and PI3K-Akt (*p* < 0.0001) (Figure 3). In addition, enhancement was associated with mTOR signalling (*p* < 0.0001), MAPK (*p* = 0.0004) signalling, Focal adhesion (*p <* 0.0001) and Apoptosis (*p* = 0.0069). Necrosis was associated with PI3K-Akt signalling (*p* = 0.0005) (Figure 3), MAPK signalling (*p* = 0.0233), Wnt signalling (*p* = 0.0054), and p53 signalling (*p* = 0.0348). Furthermore, necrosis was significantly associated with Cell cycle (*p <* 0.0001) and Focal adhesion (*p* = 0.0470).

**Table 4:** Results of oncology-wide pathway analysis of radiogenomic associations: annotation for KEGG pathways in cancer

Imaging group	Genes in input (*n*)^b^	Genes from input available in pathway (*n*)	Genes in pathway annotation (*n*)	KEGG cancer pathway	*p*-value	*p* Bonferroni corrected
necrosis degree	55	9	124	Cell cycle	<0.0001	<0.0001
necrosis degree	55	10	346	PI3K-Akt signalling pathway	0.0000	0.0005
necrosis degree	55	6	139	Wnt signalling pathway	0.0000	0.0054
necrosis degree	55	7	259	MAPK signalling pathway	0.0002	0.0233
necrosis degree	55	4	68	p53 signalling pathway	0.0003	0.0348
necrosis degree	55	6	206	Focal adhesion	0.0004	0.0470
enhancement degree***^a^***	37	12	346	PI3K-Akt signalling pathway	<0.0001	<0.0001
enhancement degree	37	8	206	Focal adhesion	<0.0001	<0.0001
enhancement degree	37	5	60	mTOR signalling pathway	<0.0001	<0.0001
enhancement degree	37	5	61	VEGF signalling pathway	<0.0001	<0.0001
enhancement degree	37	7	259	MAPK signalling pathway	0.0000	0.0004
enhancement degree	37	4	86	Apoptosis	0.0001	0.0069

^a^We excluded enhancement pattern features. ^b^We required a minimal of 20 genes of radiogenomic associations for an imaging feature group (genes from input) for inclusion in analysis.

Supplementary Table 10 summarizes associations of imaging and individual genomic features that were found in multiple cancer types. Imaging groups (*N* = 14) comprised features (e.g. tumor size, multifocality) of both MRI and CT in various malignancies. Results included the correlations of enhancement features with *VEGF*-expression in brain tumors (glioblastoma) [158] and head-neck tumors (oral cavity SCC) [156].

## DISCUSSION

This study provided a comprehensive database of imaging-genomics associations, in which queries can be made (Supplementary Table 2). This review focussed on both imaging-genomics associations with possible clinical application per cancer subtype and oncology-wide patterns in radiophenotype-genotype relations.

### Diffuse glioma

The 2016 WHO classification for diffuse glioma in adults is largely based on *IDH1*-mutation status and 1p/19q codeletion [159]. However, biopsy-based genotyping is an invasive technique that can be unreliable due to spatial tumor heterogeneity. Imaging biomarkers reflect the whole tumor and could possibly enhance genotyping accuracy non-invasively. Compared to *IDH*-wild type, *IDH*-1/2 mutated glioma have a favourable prognosis [21–23, 160]. *IDH*-status is the top-level diagnostic stratification after histology in the WHO index 2016 [159]. Although 12 out of 15 studies identified associations between imaging and *IDH*-status, the majority of findings were not independently validated. MR-perfusion [27, 28] and 2-HG MR-spectroscopy parameters [30, 31, 161], however, were correlated with *IDH*-status in multiple studies and yield potential for future imaging-based *IDH*-mutation detection. The oncometabolite 2-HG is elevated in *IDH*-mutated cases and can be depicted using MR-spectroscopy [29, 162–164], although this is technically challenging due to overlap of neighbouring metabolites (GABA, glutamate and glutamine) in the spectrum. State-of-the-art MR systems generate the high-quality spectra needed for 2-HG detection, enabling clinical practice integration [165]. The codeletion of chromosome 1p19q is an early genetic event in development of oligodendroglioma associated with greater chemosensitivity and improved survival [24–26, 159]. 1p19q co-deleted tumors were repeatedly characterised as heterogeneous [36–39] with ill-defined margins [36–39]. This phenotype is possibly caused by enhanced invasiveness of 1p19q co-deleted glioma. *MGMT*-methylated glioblastoma respond better to DNA alkylating chemotherapy with improved prognosis [41, 42]. The finding that *MGMT*-methylated cases showed higher ADC-values on DWI-MRI [27, 44–47] may only be relevant for detecting non-methylated, bad-responding elderly patients who may decide to refrain from treatment [166, 167]. *EGFR* aberrations were often correlated with MR-perfusion parameters, possibly due to the effect of *EGFR* on cell invasiveness and angiogenesis. However, despite the important role of *EGFR* in glioma development [168], suitable *EGFR*- targeted therapies for glioma have not been developed [169]. To ensure standardised glioma imaging features, models increasingly adopt quantitative imaging. Models allow for incorporating multidimensional parameters. Machine learning techniques are successfully adopted to optimize feature selection [34, 170, 171]. Potentially applicable models were found using quantitative features (3D texture, shape) [62], and a combination of quantitative and qualitative features (volume, haemorrhage, T1/FLAIR ratio) [59], both stratifying for survival and unique pathway activity. Additionally, prognostic models using quantitative imaging recently entered the phase of being tested in prospective setting [69, 171]. Although quantitative radiogenomic analyses showed great potential for genotyping in glioma, the vast variety of features and study designs made comparing results challenging.

### Non-small cell lung cancer

Since specific therapies are available for genomic subgroups of NSCLC, genotyping is important for directing therapy [172]. However, biopsy-based genotyping can cause treatment delay [173]. Radiogenomics may provide a reliable non-invasive tool for fast genotyping. *EGFR*-mutated [174, 175] and *ALK*-rearranged [172, 176, 177] tumors are targetable and are therefore extensively researched in radiogenomics. Repeatedly, FDG-PET was associated with *EGFR*-mutations, which may be biologically explained by the activating role of mutated *EGFR* glycolysis through AKT-signalling [178, 179]. The major studies showed a higher FDG-PET uptake for *EGFR*-mutated tumors; one of these validated their results in an independent cohort [73]. The three studies that found a lower uptake for *EGFR*-mutated cases were possibly unreliable because lower uptake was either not confirmed in multivariate analysis [76], found in metastasis only [75], or found because the comparison group had highly avid *KRAS*-cases [77]. Proportion GGO versus solid appearance on CT might be useful to differentiate genetic NSCLC-subtypes. Seemingly, wild type tumors have a large proportion GGO, *EGFR*-mutated tumors have a small component GGO, and *ALK*-rearranged tumors are the most solid. However, validation studies with standardised GGO measurements are needed to reliably discriminate genotypes. Similarly, standardised tumor morphology features need to be assessed in order to validate the predictive value of ill-defined tumor borders for *ALK*-status. Multiparametric (quantitative imaging) studies can be powerful for predicting individual genetic traits, as well as gene clusters related to prognosis. However, findings need to be validated in independent cohorts before they can be used in clinical practice.

### Breast cancer

In breast cancer, most radiogenomic associations were not independently validated. Limited results indicate FDG-PET can possibly discriminate molecular subtypes [106, 107]. Gene-expression scores such as Oncotype Dx recurrence risk test and MammaPrint metastasis risk test become increasingly important for clinical decision making in breast cancer, especially to prevent unnecessary chemotherapy. Since genetic tests are costly and time-consuming, studies aimed at finding imaging surrogates. In multiple studies perfusion features showed potential for predicting high-risk genetic tests, indicating tumor perfusion may be sign of poor prognosis in breast cancer. Studies furthermore indicated the potential of perfusion imaging for predicting gene expression markers for anti-VEGF treatment response. However, clinically applicable models are yet to be established.

### Colorectal carcinoma, renal cell carcinoma, hepatocellular carcinoma

In colorectal carcinoma, *KRAS*-mutation indicates irresponsiveness to *EGFR*-targeted treatment [180, 181] and showed high FDG-PET uptake in multiple studies. The lack of this association in one study [134] and a reported low accuracy for prediction [129] might be explained by false-positive high uptake due to inflammation [136]. Although findings are not yet prospectively validated, FDG-PET has great potential for providing biomarkers for *EGFR*-treatment decision making in CRC. In renal cell carcinoma, the amount of calcifications shows potential for predicting *BAP1*-status, which could be useful for assessing stage, grade and invasiveness [146, 147]. However, findings need validation. Although multiparametric modelling studies in RCC were limited, great strides are put in assessing its application for predicting prognosis- and complication risk [142, 143, 145]. For hepatocellular carcinoma, radiogenomic biomarkers could aid both treatment selection (*VEGF*-targeted and doxorubicin treatment) as well as prognosis prediction. Microscopic venous invasion, a sign of poor prognosis and high recurrence risk, was associated with small intratumoral arteries (CT), which was independently validated [182]. However, it was noticed patients were not selected indiscriminately [183]. The amount of studies and their population size were too low to draw conclusions.

### Patterns in radiogenomic associations

Repeatedly found imaging-genomics associations show patterns among different neoplasms. A convincing relation was found for enhancement on imaging and *VEGF*-expression, identified in brain and head neck cancers. The same association was found in a study (not included) assessing radiogenetics using immunohistochemistry in HCC [184]. For a more profound understanding of radiogenomic relations and underlying regulatory networks, insights into the related biological process can be of considerable value. Angiogenesis was the most mentioned biologic link between imaging and genomics in glioblastoma [48, 54, 55, 57, 66, 67, 158], oligodendroglioma [56, 185], breast cancer [102, 126], oral cavity SCC [156], and RCC [138]. Angiogenesis-related genes such as *VEGF* and *EGFR* genes were compared with angiogenesis related imaging features such as perfusion and contrast enhancement. Similarly in gene pathway analysis, angiogenesis (biology) may be the link between enhancement (imaging) and VEGF-pathway-signalling (genomics).

### Oncology-wide gene pathway analysis of radiogenomic associations

The importance of targeting multiple regulators in cancer pathways instead of single genes, is increasingly recognized. Genes associated with enhancement were enriched for the VEGF-signalling pathway. Similar to the association between contrast enhancement and the *VEGF* gene, enhancement may be associated with the VEGF signalling pathway due to its regulating role in angiogenesis. Imaging biomarkers for the VEGF pathway may have clinical implications as they could aid patient selection for *VEGF*-targeted treatment. *VEGF*-targeted therapy has been shown to be effective in various cancer types, including CRC, NSCLC, and breast cancer [186, 187].

Gene pathway analysis results indicated furthermore that contrast enhancement and necrosis detected with imaging reflect MAPK- and PI3K-Akt-mTOR-activity. Similarly, this could again aid patient selection in the future for MAPK- and PI3K-AKT-mTOR pathways-based targeted therapies [188–190]. An important limitation for the gene pathway analysis was, nevertheless, the heterogeneity of the imaging features within the particular imaging feature groups. To minimize this effect, specific subgroups were created such as “enhancement patterns” and “amount of enhancement”. Another important limitation for this analysis was that different types of genomics information (e.g. gene mutation vs gene expression) were described in literature. In addition to that, alterations can in principle result in either the activation or repression of the involved gene. In the performed analysis, the direction of the change, activation versus repression, could not be taken into account. Therefore, this analysis could only reveal associations.

Although evidently standardised imaging features and genetic tests are needed for further validation, results of this gene pathway analysis do reveal that oncology-wide associations between imaging groups and oncology pathways with potentially clinical value may exist. Our findings do not only indicate radiophenotype-genotype associations could be similar in different cancer types, but also imply that radiogenomics could aid patient selection and monitoring of pathway-targeted treatment in the future.

### Radiogenomics techniques

Different approaches were seen for conducting radiogenomics analysis. A considerable disadvantage of qualitative imaging assessment is the poor interobserver agreement. A powerful tool to overcome this is a validated feature set, such as VASARI [191] for glioma imaging [58, 60, 64, 192]. The rapidly rising capacity of quantitatively computer-extracted imaging (perfusion-, diffusion- and texture features) enables more powerful and robust prediction of genomic traits. This radiogenomic approach has proven to be powerful for prognosis-prediction [58–64, 97, 99, 142], and for revealing differential pathway-activity [60, 148, 193, 194]. The trend in radiogenomics is increasingly headed towards models of multiparametric multilevel (clinical, radiological and histopathological) data, unravelling radiogenomic networks [58, 63, 64, 105, 116]. Methodologically, however, the use of quantitative imaging is still developing. Reproducibility of quantitative parameters is a major concern, since they are highly dependent on scanner systems and software packages. Particularly in MRI, it remains challenging, as it has less standardised quantitative values compared with CT- or PET-imaging. Moreover, overfitting of data models can be an issue. Standardised datasets such as The Cancer Imaging Archive (TCIA) [195] and The Cancer Genome Atlas (TGCA) [196] can provide a solution for validation in an independent cohort. Standardization of methods and prospective validation are needed before quantitative radiogenomics can be treatment informative.

### Limitations

A limitation of this study was the marked heterogeneity of genomic and imaging features and the variety of analysing methods which made data integration challenging. Another constraint was that the effect size and the direction of associations were not always reported. There might have been publication bias for significant findings, but the novelty of this field of research reduces this risk. A limitation was that data of included multiparametric modelling studies were usually not published online, so these *p*-values could not be incorporated in the database.

### Potential of radiogenomics

Radiogenomic genotyping has the advantage that it can capture tumor heterogeneity, can be performed repeatedly for treatment monitoring, and can be performed in malignancies for which biopsy is not available. Moreover, radiogenomics is cost-effective using routine clinical imaging for analysis. The gene pathway analysis in this study revealed imaging-genomic networks in oncology and indicated that radiogenomics may be suitable for predicting efficacy of pathway-targeted therapies. Although an extensive amount of potentially valuable radiogenomic biomarkers was identified, validation studies are needed since the robustness of features obtained by different scanners remains an important concern. This study provides an extensive database of imaging-genomic associations that can guide future research to developing radiogenomic tools for treatment selection and prognosis prediction in human oncology. Radiogenomics, connecting multiparametric quantitative imaging with genomic data, yields great potential for non-invasive genotyping, thereby contributing to the shift towards precision medicine in oncology.

## METHODS

We performed this study according to the Preferred Reporting Items for Systematic Reviews and Meta-Analyses (PRISMA) statement [197].

### Search strategy and article selection

We systematically searched the Medline and Embase databases for English literature published until 1-2-2017 on radiogenomics in oncology with search terms referring to radiogenomics and oncology (Supplementary Table 1). References of included articles and literature reviews were checked for additional eligible studies. The following inclusion criteria were adopted: (1) the population consisted of human cancer patients; (2) the article comprised statistically assessed associations between imaging features on CT, MRI, FDG-PET or mammography and genomics; and (3) full-text was available in English. We excluded studies performing radiogenetics using immunohistochemistry analysis. We excluded case reports, editorial letters, and reviews.

### Extraction of study characteristics, quality checklist

Study characteristics, quality assessment, *p*-values for associations and effect measures were incorporated in a database (Supplementary Table 2). *P*-values of studies using an extensive amount of quantitative imaging features or multiparametric models were reviewed separately. For quality assessment, the QUADAS-2 checklist [198] was used, with additional items to address radiogenomics specifically, including the availability of an independent validation cohort. All data generated or analysed during this study are included in this published article (and its Supplementary Information files).

### Oncology-wide gene pathway analysis

Gene pathway analysis was performed to examine concordance between grouped radiophenotypes oncology-wide and gene pathways. For this analysis, imaging features were classified for 14 coherent imaging groups. Significant radiogenomic associations for each imaging group were selected. Only single genes were selected (e.g. no chromosome-type aberrations), and the genes were annotated according to the HUGO Gene Nomenclature Committee (HGNC) nomenclature, regardless of neoplasm location or type of genetic information (DNA mutation, gene expression (mRNA), methylation status). A minimum of 20 genes per imaging feature group was required for inclusion in the analysis. Gene pathway analysis was performed by comparing significantly associated genes within a particular imaging group with already existing functional gene pathway annotations; the cancer gene pathways in the Kyoto Encyclopaedia of Genes and Genomes (KEGG) database [193, 199]. The ToppGene Suite software (Division of Biomedical Informatics, Cincinnati Children's Hospital Medical Center, Cincinatti, OH; https://toppgene.cchmc.org/)) was used for gene pathway analysis based on functional annotation, calculating *p*-values using the hypergeometric probability mass function. method. *P*-values were corrected for multiple testing using the Bonferroni method (cutoff value 0.05).

## SUPPLEMENTARY MATERIALS TABLES























## References

[1] Kuo MD, Jamshidi N (2014). Behind the Numbers : Decoding Molecular Phenotypes ciples and Technical Considerations 1. Radiology.

[2] Rutman AM, Kuo MD (2009). Radiogenomics : Creating a link between molecular diagnostics and diagnostic imaging. Eur J Radiol.

[3] Mazurowski MA (2015). Radiogenomics : What It Is and Why It Is Important. J Am Coll Radiol.

[4] Gerlinger M, Rowan AJ, Horswell S, Larkin J, Endesfelder D, Gronroos E, Martinez P, Matthews N, Stewart A, Tarpey P, Varela I, Phillimore B, Begum S (2012). Intratumor heterogeneity and branched evolution revealed by multiregion sequencing. N Engl J Med.

[5] Patel AP, Tirosh I, Trombetta JJ, Shalek AK, Gillespie SM, Wakimoto H, Cahill DP, Nahed BV, Curry WT, Martuza RL, Louis DN, Rozenblatt-Rosen O, Suva ML (2014). Single-cell RNA-seq highlights intratumoral heterogeneity in primary glioblastoma. Science.

[6] Suzuki H, Aoki K, Chiba K, Sato Y, Shiozawa Y, Shiraishi Y, Shimamura T, Niida A, Motomura K, Ohka F, Yamamoto T, Tanahashi K, Ranjit M (2015). Mutational landscape and clonal architecture in grade II and III gliomas. Nat Genet.

[7] Hu LS, Ning S, Eschbacher JM, Baxter LC, Gaw N, Ranjbar S, Plasencia J, Dueck AC, Peng S, Smith KA, Nakaji P, Karis JP, Quarles CC (2017). Radiogenomics to characterize regional genetic heterogeneity in glioblastoma. Neuro Oncol.

[8] Molina D, Pérez-Beteta J, Luque B, Arregui E, Calvo M, Borrás JM, López C, Martino J, Velasquez C, Asenjo B, Benavides M, Herruzo I, Martínez-González A (2016). Tumour heterogeneity in glioblastoma assessed by MRI texture analysis: a potential marker of survival. Br J Radiol.

[9] Kickingereder P, Götz M, Muschelli J, Wick A, Neuberger U, Shinohara RT, Sill M, Nowosielski M, Schlemmer HP, Radbruch A, Wick W, Bendszus M, Maier-Hein KH, Bonekamp D (2016). Large-scale Radiomic Profiling of Recurrent Glioblastoma Identifies an Imaging Predictor for Stratifying Anti-Angiogenic Treatment Response. Clin Cancer Res.

[10] Grimm LJ (2016). Breast MRI radiogenomics: Current status and research implications. J Magn Reson Imaging.

[11] Bai HX, Lee AM, Yang L, Zhang P, Davatzikos C, Maris JM, Diskin SJ (2016). Imaging genomics in cancer research: limitations and promises. Br J Radiol.

[12] Moton S, Elbanan M, Zinn PO, Colen RR (2015). Imaging Genomics of Glioblastoma: Biology, Biomarkers, and Breakthroughs. Top Magn Reson Imaging.

[13] Zinn PO, Colen RR (2013). Imaging genomic mapping in glioblastoma. Neurosurgery.

[14] Zinn PO, Mahmood Z, Elbanan MG, Colen RR (2015). Imaging Genomics in Gliomas. Cancer J.

[15] ElBanan MG, Amer AM, Zinn PO, Colen RR (2015). Imaging genomics of Glioblastoma: state of the art bridge between genomics and neuroradiology. Neuroimaging Clin N Am.

[16] Goyen M (2014). Radiogenomic imaging-linking diagnostic imaging and molecular diagnostics. World J Radiol.

[17] Kuo MD, Jamshidi N (2014). Behind the numbers: Decoding molecular phenotypes with radiogenomics—guiding principles and technical considerations. Radiology.

[18] Rutman AM, Kuo MD (2009). Radiogenomics: Creating a link between molecular diagnostics and diagnostic imaging. Eur J Radiol.

[19] Zinn PO, Colen RR (2013). Imaging Genomic Mapping in Glioblastoma. Neurosurgery.

[20] Louis DN, Oghaki H, Wriestler O, Cavenee WK (2016). World Health Organization Histological Classification of Tumours of the Central Nervous System.

[21] Yan H, Parsons DW, Jin G, McLendon R, Rasheed BA, Yuan W, Kos I, Batinic-Haberle I, Jones S, Riggins GJ, Friedman H, Friedman A, Reardon D (2009). IDH1 and IDH2 mutations in gliomas. N Engl J Med.

[22] Cheng HB, Yue W, Xie C, Zhang RY, Hu SS, Wang Z (2013). IDH1 mutation is associated with improved overall survival in patients with glioblastoma: a meta-analysis. Tumour Biol.

[23] Weller M, Felsberg J, Hartmann C, Berger H, Steinbach JP, Schramm J, Westphal M, Schackert G, Simon M, Tonn JC, Heese O, Krex D, Nikkhah G (2009). Molecular predictors of progression-free and overall survival in patients with newly diagnosed glioblastoma: a prospective translational study of the German Glioma Network. J Clin Oncol.

[24] Lassman AB, Iwamoto FM, Cloughesy TF, Aldape KD, Rivera AL, Eichler AF, Louis DN, Paleologos NA, Fisher BJ, Ashby LS, Cairncross JG, Roldán GB, Wen PY (2011). International retrospective study of over 1000 adults with anaplastic oligodendroglial tumors. Neuro Oncol.

[25] G Berkey B, Shaw E, Jenkins R, Scheithauer B, Brachman D, Buckner J, Fink K, Souhami L, Laperierre N, Mehta M, Curran W (2006). Phase III trial of chemotherapy plus radiotherapy compared with radiotherapy alone for pure and mixed anaplastic oligodendroglioma: Intergroup Radiation Therapy Oncology Group Trial 9402. J Clin Oncol.

[26] van den Bent MJ, Brandes AA, Taphoorn MJ, Kros JM, Kouwenhoven MC, Delattre JY, Bernsen HJ, Frenay M, Tijssen CC, Grisold W, Sipos L, Enting RH, French PJ (2013). Adjuvant procarbazine, lomustine, and vincristine chemotherapy in newly diagnosed anaplastic oligodendroglioma: long-term follow-up of EORTC brain tumor group study 26951. J Clin Oncol.

[27] Yamashita K, Hiwatashi A, Togao O, Kikuchi K, Hatae R, Yoshimoto K, Mizoguchi M, Suzuki SO, Yoshiura T, Honda H (2016). MR Imaging-Based Analysis of Glioblastoma Multiforme: Estimation of IDH1 Mutation Status. AJNR Am J Neuroradiol.

[28] Kickingereder P, Sahm F, Radbruch A, Wick W, Heiland S, Deimling Av, Bendszus M, Wiestler B (2015). IDH mutation status is associated with a distinct hypoxia/angiogenesis transcriptome signature which is non-invasively predictable with rCBV imaging in human glioma. Sci Rep.

[29] Choi C, Ganji SK, DeBerardinis RJ, Hatanpaa KJ, Rakheja D, Kovacs Z, Yang XL, Mashimo T, Raisanen JM, Marin-Valencia I, Pascual JM, Madden CJ, Mickey BE (2012). 2-hydroxyglutarate detection by magnetic resonance spectroscopy in IDH-mutated patients with gliomas. Nat Med.

[30] Pope WB, Prins RM, Albert Thomas M, Nagarajan R, Yen KE, Bittinger MA, Salamon N, Chou AP, Yong WH, Soto H, Wilson N, Driggers E, Jang HG (2012). Non-invasive detection of 2-hydroxyglutarate and other metabolites in IDH1 mutant glioma patients using magnetic resonance spectroscopy. J Neurooncol.

[31] Kalinina J, Carroll A, Wang L, Yu Q, Mancheno DE, Wu S, Liu F, Ahn J, He M, Mao H, Van Meir EG (2012). Detection of “oncometabolite” 2-hydroxyglutarate by magnetic resonance analysis as a biomarker of IDH1/2 mutations in glioma. J Mol Med.

[32] Carrillo JA, Lai A, Nghiemphu PL, Kim HJ, Phillips HS, Kharbanda S, Moftakhar P, Lalaezari S, Yong W, Ellingson BM, Cloughesy TF, Pope WB (2012). Relationship between tumor enhancement, edema, IDH1 mutational status, MGMT promoter methylation, and survival in glioblastoma. AJNR Am J Neuroradiol.

[33] Yu J, Shi Z, Lian Y, Li Z, Liu T, Gao Y, Wang Y, Chen L, Mao Y (2016). Noninvasive IDH1 mutation estimation based on a quantitative radiomics approach for grade II glioma. European Radiology.

[34] Zhang B, Chang K, Ramkissoon S, Tanguturi S, Bi WL, Reardon DA, Ligon KL, Alexander BM, Wen PY, Huang RY (2017). Multimodal MRI features predict isocitrate dehydrogenase genotype in high-grade gliomas. Neuro Oncol.

[35] Lee S, Choi SH, Ryoo I, Yoon TJ, Kim TM, Lee SH, Park CK, Kim JH, Sohn CH, Park SH, Kim IH (2015). Evaluation of the microenvironmental heterogeneity in high-grade gliomas with IDH1/2 gene mutation using histogram analysis of diffusion-weighted imaging and dynamic-susceptibility contrast perfusion imaging. J Neurooncol.

[36] Jenkinson MD, du Plessis DG, Smith TS, Joyce KA, Warnke PC, Walker C (2006). Histological growth patterns and genotype in oligodendroglial tumours: correlation with MRI features. Brain.

[37] Kim JW, Park CK, Park SH, Kim YH, Han JH, Kim CY, Sohn CH, Chang KH, Jung HW (2011). Relationship between radiological characteristics and combined 1p and 19q deletion in World Health Organization grade III oligodendroglial tumours. J Neurol Neurosurg Psychiatry.

[38] Megyesi JF, Kachur E, Lee DH, Zlatescu MC, Betensky RA, Forsyth PA, Okada Y, Sasaki H, Mizoguchi M, Louis DN, Cairncross JG (2004). Imaging correlates of molecular signatures in oligodendrogliomas. Clin Cancer Res.

[39] Johnson DR, Diehn FE, Giannini C, Jenkins RB, Jenkins SM, Parney IF, Kaufmann TJ (2017). Genetically Defined Oligodendroglioma Is Characterized by Indistinct Tumor Borders at MRI. AJNR Am J Neuroradiol.

[40] Chawla S, Krejza J, Vossough A, Zhang Y, Kapoor GS, Wang S, O’Rourke DM, Melhem ER, Poptani H (2013). Differentiation between oligodendroglioma genotypes using dynamic susceptibility contrast perfusion-weighted imaging and proton MR spectroscopy. AJNR Am J Neuroradiol.

[41] Hegi ME, Liu L, Herman JG, Stupp R, Wick W, Weller M, Mehta MP, Gilbert MR (2008). Correlation of O6-methylguanine methyltransferase (MGMT) promoter methylation with clinical outcomes in glioblastoma and clinical strategies to modulate MGMT activity. J Clin Oncol.

[42] Hegi ME, Diserens AC, Gorlia T, Hamou MF, de Tribolet N, Weller M, Kros JM, Hainfellner JA, Mason W, Mariani L, Bromberg JE, Hau P, Mirimanoff RO (2005). MGMT gene silencing and benefit from temozolomide in glioblastoma. N Engl J Med.

[43] Stupp R, Hegi ME, Mason WP, van den Bent MJ, Taphoorn MJ, Janzer RC, Ludwin SK, Allgeier A, Fisher B, Belanger K, Hau P, Brandes AA, Gijtenbeek J (2009). Effects of radiotherapy with concomitant and adjuvant temozolomide versus radiotherapy alone on survival in glioblastoma in a randomised phase III study: 5-year analysis of the EORTC-NCIC trial. Lancet Oncol.

[44] Moon WJ, Choi JW, Roh HG, Lim SD, Koh YC (2012). Imaging parameters of high grade gliomas in relation to the MGMT promoter methylation status: the CT, diffusion tensor imaging, and perfusion MR imaging. Neuroradiology.

[45] Romano A, Calabria LF, Tavanti F, Minniti G, Rossi-Espagnet MC, Coppola V, Pugliese S, Guida D, Francione G, Colonnese C, Fantozzi LM, Bozzao A (2013). Apparent diffusion coefficient obtained by magnetic resonance imaging as a prognostic marker in glioblastomas: Correlation with MGMT promoter methylation status. Eur Radiol.

[46] Rundle-Thiele D, Day B, Stringer B, Fay M, Martin J, Jeffree RL, Thomas P, Bell C, Salvado O, Gal Y, Coulthard A, Crozier S, Rose S (2015). Using the apparent diffusion coefficient to identifying MGMT promoter methylation status early in glioblastoma: importance of analytical method. J Med Radiat Sci.

[47] Sunwoo L, Choi SH, Park CK, Kim JW, Yi KS, Lee WJ, Yoon TJ, Song SW, Kim JE, Kim JY, Kim TM, Lee SH, Kim JH (2013). Correlation of apparent diffusion coefficient values measured by diffusion MRI and MGMT promoter methylation semiquantitatively analyzed with MS-MLPA in patients with glioblastoma multiforme. J Magn Reson Imaging.

[48] Ahn SS, Shin NY, Chang JH, Kim SH, Kim EH, Kim DW, Lee SK (2014). Prediction of methylguanine methyltransferase promoter methylation in glioblastoma using dynamic contrast-enhanced magnetic resonance and diffusion tensor imaging. J Neurosurg.

[49] Drabycz S, Roldán G, de Robles P, Adler D, McIntyre JB, Magliocco AM, Cairncross JG, Mitchell JR (2010). An analysis of image texture, tumor location, and MGMT promoter methylation in glioblastoma using magnetic resonance imaging. Neuroimage.

[50] Ellingson BM, Cloughesy TF, Pope WB, Zaw TM, Phillips H, Lalezari S, Nghiemphu PL, Ibrahim H, Naeini KM, Harris RJ, Lai A (2012). Anatomic localization of O6-methylguanine DNA methyltransferase (MGMT) promoter methylated and unmethylated tumors: a radiographic study in 358 de novo human glioblastomas. Neuroimage.

[51] Ellingson BM, Lai A, Harris RJ, Selfridge JM, Yong WH, Das K, Pope WB, Nghiemphu PL, Vinters HV, Liau LM, Mischel PS, Cloughesy TF (2013). Probabilistic radiographic atlas of glioblastoma phenotypes. AJNR Am J Neuroradiol.

[52] Levner I, Drabycz S, Roldan G, De Robles P, Cairncross JG, Mitchell R (2009). Predicting MGMT methylation status of glioblastomas from MRI texture. Med Image Comput Comput Assist Interv.

[53] Young RJ, Gupta A, Shah AD, Graber JJ, Schweitzer AD, Prager A, Shi W, Zhang Z, Huse J, Omuro AM (2013). Potential role of preoperative conventional MRI including diffusion measurements in assessing epidermal growth factor receptor gene amplification status in patients with glioblastoma. AJNR Am J Neuroradiol.

[54] Gupta A, Young RJ, Shah AD, Schweitzer AD, Graber JJ, Shi W, Zhang Z, Huse J, Omuro AM (2015). Pretreatment Dynamic Susceptibility Contrast MRI Perfusion in Glioblastoma: Prediction of EGFR Gene Amplification. Clin Neuroradiol.

[55] Tykocinski ES, Grant RA, Kapoor GS, Krejza J, Bohman LE, Gocke TA, Chawla S, Halpern CH, Lopinto J, Melhem ER, O’Rourke DM (2012). Use of magnetic perfusion-weighted imaging to determine epidermal growth factor receptor variant III expression in glioblastoma. Neuro Oncol.

[56] Kapoor GS, Gocke TA, Chawla S, Whitmore RG, Nabavizadeh A, Krejza J, Lopinto J, Plaum J, Maloney-Wilensky E, Poptani H, Melhem ER, Judy KD, O’Rourke DM (2009). Magnetic resonance perfusion-weighted imaging defines angiogenic subtypes of oligodendroglioma according to 1p19q and EGFR status. J Neurooncol.

[57] Yoo RE, Choi SH, Cho HR, Kim TM, Lee SH, Park CK, Park SH, Kim IH, Yun TJ, Kim JH, Sohn CH, Han MH, Chang KH (2013). Tumor blood flow from arterial spin labeling perfusion MRI: A key parameter in distinguishing high-grade gliomas from primary cerebral lymphomas, and in predicting genetic biomarkers in high-grade gliomas. J Magn Reson Imaging.

[58] Colen RR, Vangel M, Wang J, Gutman DA, Hwang SN, Wintermark M, Jain R, Jilwan-Nicolas M, Chen JY, Raghavan P, Holder CA, Rubin D, Huang E (2014). Imaging genomic mapping of an invasive MRI phenotype predicts patient outcome and metabolic dysfunction: a TCGA glioma phenotype research group project. BMC Med Genomics.

[59] Rao A, Rao G, Gutman DA, Flanders AE, Hwang SN, Rubin DL, Colen RR, Zinn PO, Jain R, Wintermark M, Kirby JS, Jaffe CC (2016). A combinatorial radiographic phenotype may stratify patient survival and be associated with invasion and proliferation characteristics in glioblastoma. J Neurosurg.

[60] Gevaert O, Mitchell LA, Achrol AS, Xu J, Echegaray S, Steinberg GK, Cheshier SH, Napel S, Zaharchuk G, Plevritis SK (2014). Glioblastoma multiforme: exploratory radiogenomic analysis by using quantitative image features. Radiology.

[61] Diehn M, Nardini C, Wang DS, McGovern S, Jayaraman M, Liang Y, Aldape K, Cha S, Kuo MD (2008). Identification of noninvasive imaging surrogates for brain tumor gene-expression modules. Proc Natl Acad Sci U S A.

[62] Itakura H, Achrol AS, Mitchell LA, Loya JJ, Liu T, Westbroek EM, Feroze AH, Rodriguez S, Echegaray S, Azad TD, Yeom KW, Napel S, Rubin DL (2015). Magnetic resonance image features identify glioblastoma phenotypic subtypes with distinct molecular pathway activities. Sci Transl Med.

[63] Zinn PO, Sathyan P, Mahajan B, Bruyere J, Hegi M, Majumder S, Colen RR (2012). A novel volume-age-KPS (VAK) glioblastoma classification identifies a prognostic cognate microRNA-gene signature. PLoS One.

[64] Jain R, Poisson LM, Gutman D, Scarpace L, Hwang SN, Holder CA, Wintermark M, Rao A, Colen RR, Kirby J, Freymann J, Jaffe CC, Mikkelsen T (2014). Outcome prediction in patients with glioblastoma by using imaging, clinical, and genomic biomarkers: focus on the nonenhancing component of the tumor. Radiology.

[65] Grossmann P, Gutman DA, Dunn WD, Holder CA, Aerts HJ (2016). Imaging-genomics reveals driving pathways of MRI derived volumetric tumor phenotype features in Glioblastoma. BMC Cancer.

[66] Jain R, Poisson L, Narang J, Scarpace L, Rosenblum ML, Rempel S, Mikkelsen T (2012). Correlation of perfusion parameters with genes related to angiogenesis regulation in glioblastoma: a feasibility study. AJNR Am J Neuroradiol.

[67] Barajas RF, Hodgson JG, Chang JS, Vandenberg SR, Yeh RF, Parsa AT, McDermott MW, Berger MS, Dillon WP, Cha S (2010). Glioblastoma multiforme regional genetic and cellular expression patterns: influence on anatomic and physiologic MR imaging. Radiology.

[68] Rao A, Manyam G, Rao G, Jain R (2016). Integr ative Analysis of mRNA, microRNA, and Protein Correlates of Relative Cerebral Blood Volume Values in GBM Reveals the Role for Modulators of Angiogenesis and Tumor Proliferation. Cancer Inform.

[69] Heiland DH, Demerath T, Kellner E, Kiselev VG, Pfeifer D, Schnell O, Staszewski O, Urbach H, Weyerbrock A, Mader I (2016). Molecular differences between cerebral blood volume and vessel size in glioblastoma multiforme. Oncotarget.

[70] Mak RH, Digumarthy SR, Muzikansky A, Engelman JA, Shepard JAO, Choi NC, Sequist LV (2011). Role of F-18-Fluorodeoxyglucose Positron Emission Tomography in Predicting Epidermal Growth Factor Receptor Mutations in Non-Small Cell Lung Cancer. Oncologist.

[71] Na II, Byun BH, Kim KM, Cheon GJ, Choe DH, Koh JS, Lee DY, Ryoo BY, Baek H, Lim SM, Yang SH, Kim CH, Lee JC (2010). 18F-FDG uptake and EGFR mutations in patients with non-small cell lung cancer: a single-institution retrospective analysis. Lung Cancer.

[72] Cho A, Hur J, Moon YW, Hong SR, Suh YJ, Kim YJ, Im DJ, Hong YJ, Lee HJ, Kim YJ, Shim HS, Lee JS, Kim JH (2016). Correlation between EGFR gene mutation, cytologic tumor markers, 18F-FDG uptake in non-small cell lung cancer. BMC Cancer.

[73] Guan J, Xiao NJ, Chen M, Zhou WL, Zhang YW, Wang S, Dai YM, Li L, Zhang Y, Li QY, Li XZ, Yang M, Wu HB (2016). 18F-FDG uptake for prediction EGFR mutation status in non-small cell lung cancer. Medicine (Baltimore).

[74] Qiang G, Huang W, Liang C, Xu R, Yan J, Xu Y, Wang Y, Da J, Shi B, Guo Y, Liu D (2016). Association between histopathological subtype, 18F-fluorodeoxyglucose uptake and epidermal growth factor receptor mutations in lung adenocarcinoma. Oncol Lett.

[75] Yuen E, Lee P, Khong P, Radiology F, Ho V, Lee F, Radiology F, Qian W, Yu X, Wong MP (2015). Metabolic Phenotype of Stage IV Lung Adenocarcinoma Relationship With Epidermal Growth Factor Receptor Mutation. Clin Nucl Med.

[76] Lee SM, Bae SK, Jung SJ, Kim CK (2015). FDG uptake in non-small cell lung cancer is not an independent predictor of EGFR or KRAS mutation status: a retrospective analysis of 206 patients. Clin Nucl Med.

[77] Caicedo C, Garcia-Velloso MJ, Lozano MD, Labiano T, Vigil Diaz C, Lopez-Picazo JM, Gurpide A, Zulueta JJ, Zulueta J, Richter Echevarria JA, Perez Gracia JL (2014). Role of [18F]FDG PET in prediction of KRAS and EGFR mutation status in patients with advanced non-small-cell lung cancer. Eur J Nucl Med Mol Imaging.

[78] Ko KH, Hsu HH, Huang TW, Gao HW, Shen DH, Chang WC, Hsu YC, Chang TH, Chu CM, Ho CL, Chang H (2014). Value of 18F-FDG uptake on PET/CT and CEA level to predict epidermal growth factor receptor mutations in pulmonary adenocarcinoma. Eur J Nucl Med Mol Imaging.

[79] Huang CT, Yen RF, Cheng MF, Hsu YC, Wei PF, Tsai YJ, Tsai MF, Shih JY, Yang CH, Yang PC (2010). Correlation of F-18 fluorodeoxyglucose-positron emission tomography maximal standardized uptake value and EGFR mutations in advanced lung adenocarcinoma. Med Oncol.

[80] Kim TJ, Lee CT, Jheon SH, Park JS, Chung JH (2016). Radiologic Characteristics of Surgically Resected Non-Small Cell Lung Cancer With ALK Rearrangement or EGFR Mutations. Ann Thorac Surg.

[81] Chung HW, Lee KY, Kim HJ, Kim WS, So Y (2014). FDG PET/CT metabolic tumor volume and total lesion glycolysis predict prognosis in patients with advanced lung adenocarcinoma. J Cancer Res Clin Oncol.

[82] Putora PM, Fruh M, Muller J (2013). FDG-PET SUV-max values do not correlate with epidermal growth factor receptor mutation status in lung adenocarcinoma. Respirology.

[83] Hong SJ, Kim TJ, Choi YW, Park JS, Chung JH, Lee KW (2016). Radiogenomic correlation in lung adenocarcinoma with epidermal growth factor receptor mutations: Imaging features and histological subtypes. Eur Radiol.

[84] Hsu KH, Chen KC, Yang TY, Yeh YC, Chou TY, Chen HY, Tsai CR, Chen CY, Hsu CP, Hsia JY, Chuang CY, Tsai YH, Chen KY (2011). Epidermal growth factor receptor mutation status in stage I lung adenocarcinoma with different image patterns. J Thorac Oncol.

[85] Yang Y, Yang Y, Zhou X, Song X, Liu M, He W, Wang H, Wu C, Fei K, Jiang G (2015). EGFR L858R mutation is associated with lung adenocarcinoma patients with dominant ground-glass opacity. Lung Cancer.

[86] Sabri A, Batool M, Xu Z, Bethune D, Abdolell M, Manos D (2016). Predicting EGFR mutation status in lung cancer: Proposal for a scoring model using imaging and demographic characteristics. Eur Radiol.

[87] Gevaert O, Echegaray S, Khuong A, Hoang CD, Shrager JB, Jensen KC, Berry GJ, Guo HH, Lau C, Plevritis SK, Rubin DL, Napel S, Leung AN (2017). Predictive radiogenomics modeling of EGFR mutation status in lung cancer. Sci Rep.

[88] Sugano M, Shimizu K, Nakano T, Kakegawa S, Miyamae Y, Kaira K, Araki T, Kamiyoshihara M, Kawashima O, Takeyoshi I (2011). Correlation between computed tomography findings and epidermal growth factor receptor and KRAS gene mutations in patients with pulmonary adenocarcinoma. Oncol Rep.

[89] Aoki T, Hanamiya M, Uramoto H, Hisaoka M, Yamashita Y, Korogi Y (2012). Adenocarcinomas with Predominant Ground-Glass Opacity. Correlation of Morphology and Molecular Biomarkers. Radiology.

[90] Chung JH, Choe G, Jheon S, Sung SW, Kim TJ, Lee KW, Lee JH, Lee CT (2009). Epidermal Growth Factor Receptor Mutation and Pathologic-Radiologic Correlation Between Multiple Lung Nodules with Ground-Glass Opacity Differentiates Multicentric Origin from Intrapulmonary Spread. J Thorac Oncol.

[91] Glynn C, Zakowski MF, Ginsberg MS (2010). Are there imaging characteristics associated with epidermal growth factor receptor and KRAS mutations in patients with adenocarcinoma of the lung with bronchioloalveolar features?. J Thorac Oncol.

[92] Hsu JS, Huang MS, Chen CY, Liu GC, Liu TC, Chong IW, Chou SH, Yang CJ (2014). Correlation between EGFR mutation status and computed tomography features in patients with advanced pulmonary adenocarcinoma. J Thorac Imaging.

[93] Rizzo S, Petrella F, Buscarino V, De Maria F, Raimondi S, Barberis M, Fumagalli C, Spitaleri G, Rampinelli C, De Marinis F, Spaggiari L, Bellomi M (2016). CT Radiogenomic Characterization of EGFR, K-RAS, and ALK Mutations in Non-Small Cell Lung Cancer. Eur Radiol.

[94] Yano M, Sasaki H, Kobayashi Y, Yukiue H, Haneda H, Suzuki E, Endo K, Kawano O, Hara M, Fujii Y (2006). Epidermal growth factor receptor gene mutation and computed tomographic findings in peripheral pulmonary adenocarcinoma. J Thorac Oncol.

[95] Zhou JY, Zheng J, Yu ZF, Xiao WB, Zhao J, Sun K, Wang B, Chen X, Jiang LN, Ding W, Zhou JY (2015). Comparative analysis of clinicoradiologic characteristics of lung adenocarcinomas with ALK rearrangements or EGFR mutations. Eur Radiol.

[96] Fukui T, Yatabe Y, Kobayashi Y, Tomizawa K, Ito S, Hatooka S, Matsuo K, Mitsudomi T (2012). Clinicoradiologic characteristics of patients with lung adenocarcinoma harboring EML4-ALK fusion oncogene. Lung Cancer.

[97] Gevaert O, Leung AN, Quon A, Rubin DL, Napel S, Xu J, Hoang CD, Xu Y, Plevritis SK (2012). Identifying Prognostic Imaging Biomarkers by Leveraging Public Gene Expression Microarray Data. Radiology.

[98] Liu Y, Kim J, Balagurunathan Y, Li Q, Garcia AL, Stringfield O, Ye Z, Gillies RJ (2016). Radiomic Features Are Associated With EGFR Mutation Status in Lung Adenocarcinomas. Clin Lung Cancer.

[99] Weiss GJ, Ganeshan B, Miles KA, Campbell DH, Cheung PY, Frank S, Korn RL (2014). Noninvasive image texture analysis differentiates K-ras mutation from pan-wildtype NSCLC and is prognostic. PLoS One.

[100] Nair VS, Gevaert O, Davidzon G, Napel S, Graves EE, Hoang CD, Shrager JB, Quon A, Rubin DL, Plevritis SK (2012). Prognostic PET 18F-FDG Uptake Imaging Features Are Associated with Major Oncogenomic Alterations in Patients with Resected Non-Small Cell Lung Cancer. Cancer Res.

[101] Nair VS, Gevaert O, Davidzon G, Plevritis SK, West R (2014). NF-κB protein expression associates with (18)F-FDG PET tumor uptake in non-small cell lung cancer: a radiogenomics validation study to understand tumor metabolism. Lung Cancer.

[102] Yoon HJ, Kang KW, Chun IK, Cho N, Im SA, Jeong S, Lee S, Jung KC, Lee YS, Jeong JM, Lee DS, Chung JK, Moon WK (2014). Correlation of breast cancer subtypes, based on estrogen receptor, progesterone receptor, and HER2, with functional imaging parameters from 68Ga-RGD PET/CT and 18F-FDG PET/CT. Eur J Nucl Med Mol Imaging.

[103] Ozkan E, West A, Dedelow JA, Chu BF, Zhao W, Yildiz VO, Otterson GA, Shilo K, Ghosh S, King M, White RD, Erdal BS (2015). CT gray-level texture analysis as a quantitative imaging biomarker of epidermal growth factor receptor mutation status in adenocarcinoma of the lung. AJR Am J Roentgenol.

[104] Aerts HJ, Grossmann P, Tan Y, Oxnard GG, Rizvi N, Schwartz LH, Zhao B (2016). Defining a Radiomic Response Phenotype: A Pilot Study using targeted therapy in NSCLC. Sci Rep.

[105] Yoon HJ, Sohn I, Cho JH, Lee HY, Kim JH, Choi YL, Kim H, Lee G, Lee KS, Kim J (2015). Decoding Tumor Phenotypes for ALK, ROS1, and RET Fusions in Lung Adenocarcinoma Using a Radiomics Approach. Medicine (Baltimore).

[106] Palaskas N, Larson SM, Schultz N, Komisopoulou E, Wong J, Rohle D, Campos C, Yannuzzi N, Osborne JR, Linkov I, Kastenhuber ER, Taschereau R, Plaisier SB (2011). 18F-fluorodeoxy-glucose positron emission tomography marks MYC-overexpressing human basal-like breast cancers. Cancer Res.

[107] Osborne JR, Port E, Gonen M, Doane A, Yeung H, Gerald W, Cook JB, Larson S (2010). 18F-FDG PET of locally invasive breast cancer and association of estrogen receptor status with standardized uptake value: microarray and immunohistochemical analysis. J Nucl Med.

[108] Mazurowski MA, Zhang J, Grimm LJ, Yoon SC, Silber JI (2014). Radiogenomic Analysis of Breast Cancer: Luminal B Molecular Subtype Is Associated with Enhancement Dynamics at MR Imaging. Radiology.

[109] Veltman J, Mann R, Kok T, Obdeijn IM, Hoogerbrugge N, Blickman JG, Boetes C (2008). Breast tumor characteristics of BRCA1 and BRCA2 gene mutation carriers on MRI. Eur Radiol.

[110] Ashraf AB, Daye D, Gavenonis S, Mies C, Feldman M, Rosen M, Kontos D (2014). Identification of intrinsic imaging phenotypes for breast cancer tumors: preliminary associations with gene expression profiles. Radiology.

[111] Siamakpour-Reihani S, Owzar K, Jiang C, Scarbrough PM, Craciunescu OI, Horton JK, Dressman HK, Blackwell KL, Dewhirst MW (2015). Genomic profiling in locally advanced and inflammatory breast cancer and its link to DCE-MRI and overall survival. Int J Hyperthermia.

[112] Sutton EJ, Oh JH, Dashevsky BZ, Veeraraghavan H, Apte AP, Thakur SB, Deasy JO, Morris EA (2015). Breast cancer subtype intertumor heterogeneity: MRI-based features predict results of a genomic assay. J Magn Reson Imaging.

[113] Yamamoto S, Han W, Kim Y, Du L, Jamshidi N, Huang D, Kim JH, Kuo MD (2015). Breast Cancer: Radiogenomic Biomarker Reveals Associations among Dynamic Contrast-enhanced MR Imaging, Long Noncoding RNA, and Metastasis. Radiology.

[114] Yamamoto S, Maki DD, Korn RL, Kuo MD (2012). Radiogenomic Analysis of Breast Cancer Using MRI: A Preliminary Study to Define the Landscape. AJR Am J Roentgenol.

[115] Fernandez-Navarro P, González-Neira A, Pita G, Díaz-Uriarte R, Tais Moreno L, Ederra M, Pedraz-Pingarrón C, Sánchez-Contador C, Vázquez-Carrete JA, Moreo P, Vidal C, Salas-Trejo D, Stone J (2015). Genome wide association study identifies a novel putative mammographic density locus at 1q12-q21. Int J Cancer.

[116] Zhu Y, Li H, Guo W, Drukker K, Lan L, Giger ML, Ji Y (2015). Deciphering Genomic Underpinnings of Quantitative MRI-based Radiomic Phenotypes of Invasive Breast Carcinoma. Sci Rep.

[117] Li H, Giger ML, Sun C, Ponsukcharoen U, Huo D, Lan L, Olopade OI, Jamieson AR, Brown JB, Di Rienzo A (2014). Pilot study demonstrating potential association between breast cancer image-based risk phenotypes and genomic biomarkers. Med Phys.

[118] Li H, Zhu Y, Burnside ES, Drukker K, Hoadley KA, Fan C, Conzen SD, Whitman GJ, Sutton EJ, Net JM, Ganott M, Huang E, Morris EA (2016). MR Imaging Radiomics Signatures for Predicting the Risk of Breast Cancer Recurrence as Given by Research Versions of MammaPrint, Oncotype DX, and PAM50 Gene Assays. Radiology.

[119] Mehta S, Hughes NP, Li S, Jubb A, Adams R, Lord S, Koumakis L, van Stiphout R, Padhani A, Makris A, Buffa FM, Harris AL (2016). Radiogenomics Monitoring in Breast Cancer Identifies Metabolism and Immune Checkpoints as Early Actionable Mechanisms of Resistance to Anti-angiogenic Treatment. EBioMedicine.

[120] Wan T, Bloch BN, Plecha D, Thompson CL, Gilmore H, Jaffe C, Harris L, Madabhushi A (2016). A Radio-genomics Approach for Identifying High Risk Estrogen Receptor-positive Breast Cancers on DCE-MRI: Preliminary Results in Predicting OncotypeDX Risk Scores. Sci Rep.

[121] Dialani V, Gaur S, Mehta TS, Venkataraman S, Fein-Zachary V, Phillips J, Brook A, Slanetz PJ (2016). Prediction of Low versus High Recurrence Scores in Estrogen Receptor-Positive, Lymph Node-Negative Invasive Breast Cancer on the Basis of Radiologic-Pathologic Features: Comparison with Oncotype DX Test Recurrence Scores. Radiology.

[122] Tang G, Shak S, Paik S, Anderson SJ, Costantino JP, Geyer CE, Mamounas EP, Wickerham DL, Wolmark N (2011). Comparison of the prognostic and predictive utilities of the 21-gene Recurrence Score assay and Adjuvant! for women with node-negative, ER-positive breast cancer: results from NSABP B-14 and NSABP B-20. Breast Cancer Res Treat.

[123] Kittaneh M, Montero AJ (2013). Biomarkers in Cancer Molecular Profiling for. Breast Cancer : A Comprehensive Review. Biomark Cancer.

[124] Prat A, Parker JS, Fan C, Perou CM (2012). PAM50 assay and the three-gene model for identifying the major and clinically relevant molecular subtypes of breast cancer. Breast Cancer Res Treat.

[125] Naoi Y, Noguchi S (2016). Multi-gene classifiers for prediction of recurrence in breast cancer patients. Breast Cancer.

[126] Yamamoto S, Han W, Kim Y, Du L, Jamshidi N, Huang D, Kim JH, Kuo MD (2015). Breast Cancer: Radiogenomic Biomarker Reveals Associations among Dynamic Contrast-enhanced MR Imaging, Long Noncoding RNA, and Metastasis. Radiology.

[127] Shin YR, Kim KA, Im S, Hwang SS, Kim K (2016). Prediction of KRAS Mutation in Rectal Cancer Using MRI. Anticancer Res.

[128] Chen SW, Chiang HC, Chen WTL, Hsieh TC, Yen KY, Chiang SF, Kao CH (2014). Correlation between PET/CT parameters and KRAS expression in colorectal cancer. Clin Nucl Med.

[129] Chen SW, Lin CY, Ho CM, Chang YS, Yang SF, Kao CH, Chang JG (2015). Genetic Alterations in Colorectal Cancer Have Different Patterns on 18F-FDG PET/CT. Clin Nucl Med.

[130] Izuishi K, Yamamoto Y, Sano T, Takebayashi R, Nishiyama Y, Mori H, Masaki T, Morishita A, Suzuki Y (2012). Molecular mechanism underlying the detection of colorectal cancer by 18F-2-fluoro-2-deoxy-D-glucose positron emission tomography. J Gastrointest Surg.

[131] Shi S, Ji S, Qin Y, Xu J, Zhang B, Xu W, Liu J, Long J, Liu C, Liu L, Ni Q, Yu X (2015). Metabolic tumor burden is associated with major oncogenomic alterations and serum tumor markers in patients with resected pancreatic cancer. Cancer Lett.

[132] Kawada K, Nakamoto Y, Kawada M, Hida K, Matsumoto T, Murakami T, Hasegawa S, Togashi K, Sakai Y (2012). Relationship between 18F-fluorodeoxyglucose accumulation and KRAS/BRAF mutations in colorectal cancer. Clin Cancer Res.

[133] Lovinfosse P, Koopmansch B, Lambert F, Jodogne S, Kustermans G, Hatt M, Visvikis D, Seidel L, Polus M, Albert A, Delvenne P, Hustinx R (2016). 18F-FDG PET/CT imaging in rectal cancer: relationship with the RAS mutational status. Br J Radiol.

[134] Krikelis D, Skoura E, Kotoula V, Rondogianni P, Pianou N, Samartzis A, Xanthakis I, Fountzilas G, Datseris IE (2014). Lack of association between KRAS mutations and 18F-FDG PET/CT in Caucasian metastatic colorectal cancer patients. Anticancer Res.

[135] Miles KA, Ganeshan B, Rodriguez-Justo M, Goh VJ, Ziauddin Z, Engledow A, Meagher M, Endozo R, Taylor SA, Halligan S, Ell PJ, Groves AM (2014). Multifunctional imaging signature for V-KI-RAS2 Kirsten rat sarcoma viral oncogene homolog (KRAS) mutations in colorectal cancer. J Nucl Med.

[136] Lee JH, Kang J, Baik SH, Lee KY, Lim BJ, Jeon TJ, Ryu YH, Sohn SK (2016). Relationship Between 18F-Fluorodeoxyglucose Uptake and V-Ki-Ras2 Kirsten Rat Sarcoma Viral Oncogene Homolog Mutation in Colorectal Cancer Patients: Variability Depending on C-Reactive Protein Level. Medicine (Baltimore).

[137] Miles KA, Ganeshan B, Rodriguez-Justo M, Goh VJ, Ziauddin Z, Engledow A, Meagher M, Endozo R, Taylor SA, Halligan S, Ell PJ, Groves AM (2014). Multifunctional Imaging Signature for V-KI-RAS2 Kirsten Rat Sarcoma Viral Oncogene Homolog (KRAS) Mutations in Colorectal Cancer. J Nucl Med.

[138] Karlo CA, Di Paolo PL, Chaim J, Hakimi AA, Ostrovnaya I, Russo P, Hricak H, Motzer R, Hsieh JJ, Akin O (2014). Radiogenomics of Clear Cell Renal Cell Carcinoma: Associations between CT Imaging Features and Mutations. Radiology.

[139] Sauk SC, Hsu MS, Margolis DJ, Lu DS, Rao NP, Belldegrun AS, Pantuck AJ, Raman SS (2011). Clear cell renal cell carcinoma: multiphasic multidetector CT imaging features help predict genetic karyotypes. Radiology.

[140] Shinagare AB, Vikram R, Jaffe C, Akin O, Kirby J, Huang E, Freymann J, Sainani NI, Sadow CA, Bathala TK, Rubin DL, Oto A, Heller MT (2015). Radiogenomics of clear cell renal cell carcinoma : preliminary findings of The Cancer Genome Atlas – Renal Cell Carcinoma (TCGA – RCC) Imaging Research Group. Abdom Imaging.

[141] Young JR, Margolis D, Sauk S, Pantuck AJ, Sayre J, Young JA, Hsu M, Raman SS (2014). Clear cell renal cell carcinoma: Multiphasic MDCT enhancement can predict the loss of chromosome 8p. Abdom Imaging.

[142] Jamshidi N, Jonasch E, Zapala M, Korn RL, Aganovic L, Zhao H, Tumkur Sitaram R, Tibshirani RJ, Banerjee S, Brooks JD, Ljungberg B, Kuo MD (2015). The Radiogenomic Risk Score: Construction of a Prognostic Quantitative, Noninvasive Image-based Molecular Assay for Renal Cell Carcinoma. Radiology.

[143] Jamshidi N, Jonasch E, Zapala M, Korn RL, Brooks JD, Ljungberg B, Kuo MD (2016). The radiogenomic risk score stratifies outcomes in a renal cell cancer phase 2 clinical trial. Eur Radiol.

[144] Johannesma PC, Houweling AC, Menko FH, van de Beek I, Reinhard R, Gille JJ, van Waesberghe JT, Thunnissen E, Starink TM, Postmus PE, van Moorselaar RJ (2016). Are lung cysts in renal cell cancer (RCC) patients an indication for FLCN mutation analysis?. Fam Cancer.

[145] Zhu H, Chen H, Lin Z, Shi G, Lin X, Wu Z, Zhang X, Zhang X (2016). Identifying molecular genetic features and oncogenic pathways of clear cell renal cell carcinoma through the anatomical (PADUA) scoring system. Oncotarget.

[146] Hakimi AA, Chen Y, Wren J, Gonen M, Abdel-Wahab O, Heguy A, Liu H, Takeda S, Tickoo SK, Reuter VE, Voss MH, Motzer RJ, Coleman JA (2013). Clinical and pathologic impact of select chromatin-modulating tumor suppressors in clear cell renal cell carcinoma. Eur Urol.

[147] Kapur P, Peña-Llopis S, Christie A, Zhrebker L, Pavía-Jiménez A, Rathmell WK, Xie XJ, Brugarolas J (2015). Effects on survival of BAP1 and PBRM1 mutations in sporadic clear-cell renal cell carcinoma: a retrospective analysis with independent validation. Lancet Oncol.

[148] Segal E, Sirlin CB, Ooi C, Adler AS, Gollub J, Chen X, Chan BK, Matcuk GR, Barry CT, Chang HY, Kuo MD (2007). Decoding global gene expression programs in liver cancer by noninvasive imaging. Nat Biotechnol.

[149] Miura T, Ban D, Tanaka S, Mogushi K, Kudo A, Matsumura S, Mitsunori Y, Ochiai T, Tanaka H, Tanabe M (2015). Distinct clinicopathological phenotype of hepatocellular carcinoma with ethoxybenzyl-magnetic resonance imaging hyperintensity: Association with gene expression signature. Am J Surg.

[150] Kuo MD, Gollub J, Sirlin CB, Ooi C, Chen X (2007). Radiogenomic Analysis to Identify Imaging Phenotypes Associated with Drug Response Gene Expression Programs in Hepatocellular Carcinoma. J Vasc Interv Radiol.

[151] Llovet JM, Ricci S, Mazzaferro V, Hilgard P, Gane E, Blanc JF, de Oliveira AC, Santoro A, Raoul JL, Forner A, Schwartz M, Porta C, Zeuzem S (2008). Sorafenib in Advanced Hepatocellular Carcinoma. N Engl J Med.

[152] Timmers HJ, Chen CC, Carrasquillo JA, Whatley M, Ling A, Eisenhofer G, King KS, Rao JU, Wesley RA, Adams KT, Pacak K (2012). Staging and functional characterization of pheochromocytoma and paraganglioma by 18F-fluorodeoxyglucose (18F-FDG) positron emission tomography. J Natl Cancer Inst.

[153] Blanchet EM, Gabriel S, Martucci V, Fakhry N, Chen CC, Deveze A, Millo C, Barlier A, Pertuit M, Loundou A, Pacak K, Taïeb D (2014). 18F-FDG PET/CT as a predictor of hereditary head and neck paragangliomas. Eur J Clin Invest.

[154] Venkatesan AM, Trivedi H, Adams KT, Kebebew E, Pacak K, Hughes MS (2011). Comparison of clinical and imaging features in succinate dehydrogenase-positive versus sporadic paragangliomas. Surgery.

[155] Taieb D, Sebag F, Barlier A, Tessonnier L, Palazzo FF, Morange I, Niccoli-Sire P, Fakhry N, De Micco C, Cammilleri S, Enjalbert A, Henry JF, Mundler O (2009). Avidity of Pheochromocytomas and Paragangliomas: A New Molecular Imaging Signature? J Nucl Med.

[156] Pickering CR, Shah K, Ahmed S, Rao A, Frederick MJ, Zhang J, Unruh AK, Wang J, Ginsberg LE, Kumar AJ, Myers JN, Hamilton JD (2013). CT imaging correlates of genomic expression for oral cavity squamous cell carcinoma. AJNR Am J Neuroradiol.

[157] Hoefling NL, McHugh JB, Light E, Kumar B, Walline H, Prince M, Bradford C, Carey TE, Mukherji SK (2013). Human papillomavirus, p16, and epidermal growth factor receptor biomarkers and CT perfusion values in head and neck squamous cell carcinoma. AJNR Am J Neuroradiol.

[158] Barajas RF, Phillips JJ, Vandenberg SR, McDermott MW, Berger MS, Dillon WP, Cha S (2015). Pro-angiogenic cellular and genomic expression patterns within glioblastoma influences dynamic susceptibility weighted perfusion MRI. Clin Radiol.

[159] Louis DN, Perry A, Reifenberger G, von Deimling A, Figarella-Branger D, Cavenee WK, Ohgaki H, Wiestler OD, Kleihues P, Ellison DW (2016). The 2016 World Health Organization Classification of Tumors of the Central Nervous System: a summary. Acta Neuropathol.

[160] Hartmann C, Hentschel B, Simon M, Westphal M, Schackert G, Tonn JC, Loeffler M, Reifenberger G, Pietsch T, Von Deimling A, Weller M (2013). Long-term survival in primary glioblastoma with versus without isocitrate dehydrogenase mutations. Clin Cancer Res.

[161] Choi C, Ganji SK, DeBerardinis RJ, Hatanpaa KJ, Rakheja D, Kovacs Z, Yang XL, Mashimo T, Raisanen JM, Marin-Valencia I, Pascual JM, Madden CJ, Mickey BE (2012). 2-hydroxyglutarate detection by magnetic resonance spectroscopy in IDH-mutated patients with gliomas. Nat Med.

[162] Andronesi OC, Kim GS, Gerstner E, Batchelor T, Tzika AA, Fantin VR, Vander Heiden MG, Sorensen AG (2012). Detection of 2-hydroxyglutarate in IDH-mutated glioma patients by in vivo spectral-editing and 2D correlation magnetic resonance spectroscopy. Sci Transl Med.

[163] Andronesi OC, Rapalino O, Gerstner E, Chi A, Batchelor TT, Cahill DP, Sorensen AG, Rosen BR (2013). Detection of oncogenic IDH1 mutations using magnetic resonance spectroscopy of 2-hydroxyglutarate. J Clin Invest.

[164] Lazovic J, Soto H, Piccioni D, Lou JR, Li S, Mirsadraei L, Yong W, Prins R, Liau LM, Ellingson BM, Cloughesy TF, Lai A, Pope WB (2012). Detection of 2-hydroxyglutaric acid in vivo by proton magnetic resonance spectroscopy in U87 glioma cells overexpressing isocitrate dehydrogenase-1 mutation. Neuro Oncol.

[165] De La Fuente MI, Young RJ, Rubel J, Rosenblum M, Tisnado J, Briggs S, Arevalo-Perez J, Cross JR, Campos C, Straley K, Zhu D, Dong C, Thomas A (2016). Integration of 2-hydroxyglutarate-proton magnetic resonance spectroscopy into clinical practice for disease monitoring in isocitrate dehydrogenase-mutant glioma. Neuro Oncol.

[166] Reifenberger G, Hentschel B, Felsberg J, Schackert G, Simon M, Schnell O, Westphal M, Wick W, Pietsch T, Loeffler M, Weller M (2012). German Glioma Network. Predictive impact of MGMT promoter methylation in glioblastoma of the elderly. Int J Cancer.

[167] Brandes AA, Franceschi E, Tosoni A, Benevento F, Scopece L, Mazzocchi V, Bacci A, Agati R, Calbucci F, Ermani M (2009). Temozolomide concomitant and adjuvant to radiotherapy in elderly patients with glioblastoma: Correlation with MGMT promoter methylation status. Cancer.

[168] Verhaak RG, Hoadley KA, Purdom E, Wang V, Qi Y, Wilkerson MD, Miller CR, Ding L, Golub T, Mesirov JP, Alexe G, Lawrence M, O’Kelly M (2010). Integrated genomic analysis identifies clinically relevant subtypes of glioblastoma characterized by abnormalities in PDGFRA, IDH1, EGFR, and NF1. Cancer Cell.

[169] Padfield E, Ellis HP, Kurian KM (2015). Current Therapeutic Advances Targeting EGFR and EGFRvIII in Glioblastoma. Front Oncol.

[170] Kickingereder P, Bonekamp D, Nowosielski M, Kratz A, Sill M, Burth S, Wick A, Eidel O, Schlemmer H, Radbruch A, Debus J, Herold-Mende C, Unterberg A (2016). Radiogenomics of Glioblastoma: Machine Learning-based Classification of Molecular Characteristics by Using Multiparametric and Multiregional MR Imaging Features. Radiology.

[171] Macyszyn L, Akbari H, Pisapia JM, Da X, Attiah M, Pigrish V, Bi Y, Pal S, Davuluri RV, Roccograndi L, Dahmane N, Martinez-Lage M, Biros G (2016). Imaging patterns predict patient survival and molecular subtype in glioblastoma via machine learning techniques. Neuro Oncol.

[172] Tan WL, Jain A, Takano A, Newell EW, Iyer NG, Lim W, Tan EH, Zhai W, Hillmer AM, Tam W, Tan DSW (2016). Novel therapeutic targets on the horizon for lung cancer. Lancet Oncol.

[173] Mohammed N, Kestin LL, Grills IS, Battu M, Fitch DL, Wong CY, Margolis JH, Chmielewski GW, Welsh RJ (2011). Rapid disease progression with delay in treatment of non-small-cell lung cancer. Int J Radiat Oncol Biol Phys.

[174] Rosell R, Moran T, Queralt C, Porta R, Cardenal F, Camps C, Majem M, Lopez-Vivanco G, Isla D, Provencio M, Insa A, Massuti B, Gonzalez-Larriba JL (2009). Screening for epidermal growth factor receptor mutations in lung cancer. N Engl J Med.

[175] Sequist LV, Bell DW, Lynch TJ, Haber DA (2007). Molecular predictors of response to epidermal growth factor receptor antagonists in non-small-cell lung cancer. J Clin Oncol.

[176] Shaw AT, Yeap BY, Mino-Kenudson M, Digumarthy SR, Costa DB, Heist RS, Solomon B, Stubbs H, Admane S, McDermott U, Settleman J, Kobayashi S, Mark EJ (2009). Clinical features and outcome of patients with non-small-cell lung cancer who harbor EML4-ALK. J Clin Oncol.

[177] Shaw AT, Kim DW, Nakagawa K, Seto T, Crinó L, Ahn MJ, De Pas T, Besse B, Solomon BJ, Blackhall F, Wu YL, Thomas M, O’Byrne KJ (2013). Crizotinib versus chemotherapy in advanced ALK-positive lung cancer. N Engl J Med.

[178] Buzzai M, Bauer DE, Jones RG, Deberardinis RJ, Hatzivassiliou G, Elstrom RL, Thompson CB (2005). The glucose dependence of Akt-transformed cells can be reversed by pharmacologic activation of fatty acid beta-oxidation. Oncogene.

[179] Sordella R, Bell DW, Haber DA, Settleman J (2004). Gefitinib-sensitizing EGFR mutations in lung cancer activate anti-apoptotic pathways. Science.

[180] Karapetis CS, Khambata-Ford S, Jonker DJ, O’Callaghan CJ, Tu D, Tebbutt NC, Simes RJ, Chalchal H, Shapiro JD, Robitaille S, Price TJ, Shepherd L, Au HJ (2008). K-ras mutations and benefit from cetuximab in advanced colorectal cancer. N Engl J Med.

[181] Lievre A, Bachet JB, Boige V, Cayre A, Le Corre D, Buc E, Ychou M, Bouche O, Landi B, Louvet C, Andre T, Bibeau F, Diebold MD (2008). KRAS Mutations As an Independent Prognostic Factor in Patients With Advanced Colorectal Cancer Treated With Cetuximab. J Clin Oncol.

[182] Banerjee S, Wang DS, Kim HJ, Sirlin CB, Chan MG, Korn RL, Rutman AM, Siripongsakun S, Lu D, Imanbayev G, Kuo MD (2015). A computed tomography radiogenomic biomarker predicts microvascular invasion and clinical outcomes in hepatocellular carcinoma. Hepatology.

[183] Tang H, Bai HX, Su C, Lee AM, Yang L (2016). The effect of cirrhosis on radiogenomic biomarker's ability to predict microvascular invasion and outcome in hepatocellular carcinoma. Hepatology.

[184] Kanematsu M, Osada S, Amaoka N, Goshima S, Kondo H, Moriyama N (2006). Expression of vascular endothelial growth factor in hepatocellular carcinoma and the surrounding liver: Correlation with MR imaging and angiographically assisted CT. Abdom Imaging.

[185] Narang J, Jain R, Scarpace L, Saksena S, Schultz LR, Rock JP, Rosenblum M, Patel SC, Mikkelsen T (2011). Tumor vascular leakiness and blood volume estimates in oligodendrogliomas using perfusion CT: an analysis of perfusion parameters helping further characterize genetic subtypes as well as differentiate from astroglial tumors. J Neurooncol.

[186] Goel HL, Mercurio AM (2013). VEGF targets the tumour cell. Nat Rev Cancer.

[187] Ellis LM, Hicklin DJ (2008). VEGF-targeted therapy: mechanisms of anti-tumour activity. Nat Rev Cancer.

[188] Samatar AA, Poulikakos PI (2014). Targeting RAS-ERK signalling in cancer: promises and challenges. Nat Rev Drug Discov.

[189] Porta C, Paglino C, Mosca A (2014). Targeting PI3K/Akt/mTOR Signaling in Cancer. Front Oncol.

[190] Stratikopoulos EE, Parsons RE (2016). Molecular Pathways: Targeting the PI3K Pathway in Cancer--BET Inhibitors to the Rescue. Clin Cancer Res.

[191] VASARI Research Project - The Cancer Imaging Archive (TCIA) Public Access - Cancer Imaging Archive Wiki. https://wiki.cancerimagingarchive.net/display/Public/VASARI+Research+Project.

[192] Gutman DA, Cooper LA, Hwang SN, Holder CA, Gao J, Aurora TD, Dunn WD, Scarpace L, Mikkelsen T, Jain R, Wintermark M, Jilwan M, Raghavan P (2013). MR imaging predictors of molecular profile and survival: multi-institutional study of the TCGA glioblastoma data set. Radiology.

[193] Kanehisa M, Goto S (2000). KEGG: kyoto encyclopedia of genes and genomes. Nucleic Acids Res.

[194] Halle C, Andersen E, Lando M, Aarnes EK, Hasvold G, Holden M, Syljuasen RG, Sundfor K, Kristensen GB, Holm R, Malinen E, Lyng H (2012). Hypoxia-induced gene expression in chemoradioresistant cervical cancer revealed by dynamic contrast-enhanced MRI. Cancer Res.

[195] Clark K, Vendt B, Smith K, Freymann J, Kirby J, Koppel P, Moore S, Phillips S, Maffitt D, Pringle M, Tarbox L, Prior F (2013). The Cancer Imaging Archive (TCIA): Maintaining and Operating a Public Information Repository. J Digit Imaging.

[196] The Cancer Genome Atlas-TCGA https://cancergenome.nih.gov/.

[197] Moher D, Liberati A, Tetzlaff J, Altman DG, PRISMA Group (2009). Preferred reporting items for systematic reviews and meta analyses: The Prisma Statement. PLoS Med.

[198] Whiting PF, Rutjes AW, Westwood ME, Mallett S, Deeks JJ, Reitsma JB, Leeflang MM, Sterne JA (2011). Bossuyt PM; QUADAS-2 Group. QUADAS-2: a revised tool for the quality assessment of diagnostic accuracy studies. Ann Intern Med.

[199] Kanehisa M, Sato Y, Kawashima M, Furumichi M, Tanabe M (2016). KEGG as a reference resource for gene and protein annotation. Nucleic Acids Res.

[200] Wang K, Wang Y, Fan X, Wang J, Li G, Ma J, Ma J, Jiang T, Dai J (2016). Radiological features combined with IDH1 status for predicting the survival outcome of glioblastoma patients. Neuro Oncol.

[201] Yu J, Shi Z, Ji C, Lian Y, Wang Y, Chen L (2017). Anatomical location differences between mutated and wild-type isocitrate dehydrogenase 1 in low- grade gliomas. Int J Neurosci.

[202] Wang Y, Zhang T, Li S, Fan X, Ma J, Wang L, Jiang T (2015). Anatomical localization of isocitrate dehydrogenase 1 mutation: a voxel-based radiographic study of 146 low-grade gliomas. Eur J Neurol.

[203] Metellus P, Coulibaly B, Colin C, de Paula AM, Vasiljevic A, Taieb D, Barlier A, Boisselier B, Mokhtari K, Wang XW, Loundou A, Chapon F, Pineau S (2010). Absence of IDH mutation identifies a novel radiologic and molecular subtype of WHO grade II gliomas with dismal prognosis. Acta Neuropathol.

[204] Metellus P, Colin C, Taieb D, Guedj E, Nanni-Metellus I, De Paula AM, Colavolpe C, Fuentes S, Dufour H, Barrie M, Chinot O, Ouafik L, Figarella-Branger D (2011). IDH mutation status impact on in vivo hypoxia biomarkers expression: New insights from a clinical, nuclear imaging and immunohistochemical study in 33 glioma patients. J Neurooncol.

[205] Saito T, Muragaki Y, Maruyama T, Komori T (2016). Calcification on CT is a simple and valuable preoperative indicator of 1p/19q loss of heterozygosity in supratentorial brain tumors that are suspected grade II and III gliomas. Brain Tumor Pathol.

[206] Nakae S, Murayama K, Sasaki H, Kumon M, Nishiyama Y, Ohba S, Adachi K, Nagahisa S, Hayashi T, Inamasu J, Abe M, Hasegawa M, Hirose Y (2017). Prediction of genetic subgroups in adult supra tentorial gliomas by pre- and intraoperative parameters. J Neurooncol.

[207] Fellah S, Caudal D, De Paula AM, Dory-Lautrec P, Figarella-Branger D, Chinot O, Metellus P, Cozzone PJ, Confort-Gouny S, Ghattas B, Callot V, Girard N (2013). Multimodal MR Imaging (Diffusion, Perfusion, and Spectroscopy): Is It Possible to Distinguish Oligodendroglial Tumor Grade and 1p/19q Codeletion in the Pretherapeutic Diagnosis?. AJNR Am J Neuroradiol.

[208] Sankar T, Moore NZ, Johnson J, Ashby LS, Scheck AC, Shapiro WR, Smith KA, Spetzler RF, Preul MC (2012). Magnetic resonance imaging volumetric assessment of the extent of contrast enhancement and resection in oligodendroglial tumors. J Neurosurg.

[209] Heiland DH, Mader I, Schlosser P, Pfeifer D, Carro MS, Lange T, Schwarzwald R, Vasilikos I, Urbach H, Weyerbrock A (2016). Integrative Network-based Analysis of Magnetic Resonance Spectroscopy and Genome Wide Expression in Glioblastoma multiforme. Sci Rep.

[210] Brown R, Zlatescu M, Sijben A, Roldan G, Easaw J, Forsyth P, Parney I, Sevick R, Yan E, Demetrick D, Schiff D, Cairncross G, Mitchell R (2008). The Use of Magnetic Resonance Imaging to Noninvasively Detect Genetic Signatures in Oligodendroglioma. Clin Cancer Res.

[211] Arevalo-Perez J, Thomas AA, Kaley T, Lyo J, Peck KK, Holodny AI, Mellinghoff IK, Shi W, Zhang Z, Young RJ (2015). T1-weighted dynamic contrast-enhanced MRI as a noninvasive biomarker of epidermal growth factor receptor VIII status. AJNR Am J Neuroradiol.

[212] Kanas VG, Zacharaki EI, Thomas GA, Zinn PO, Megalooikonomou V, Colen RR (2017). Learning MRI-based classification models for MGMT methylation status prediction in glioblastoma. Comput Methods Programs Biomed.

[213] Kong DS, Kim J, Lee IH, Kim ST, Seol HJ, Lee JI, Park WY, Ryu G, Wang Z, Ma’ayan A, Nam DH (2016). Integrative radiogenomic analysis for multicentric radiophenotype in glioblastoma. Oncotarget.

[214] Pope WB, Chen JH, Dong J, Carlson MR, Perlina A, Cloughesy TF, Liau LM, Mischel PS, Nghiemphu P, Lai A, Nelson SF (2008). Relationship between gene expression and enhancement in glioblastoma multiforme: exploratory DNA microarray analysis. Radiology.

[215] Raza SM, Fuller GN, Rhee CH, Huang S, Hess K, Zhang W, Sawaya R (2004). Identification of Necrosis-Associated Genes in Glioblastoma by cDNA Microarray Analysis. Clin Cancer Res.

[216] Jajamovich GH, Valiathan CR, Cristescu R (2016). Integrative analysis of diffusion weighted MRI and genomic data to inform treatment of glioblastoma. J Neurooncol.

[217] Qian X, Tan H, Zhang J, Liu K, Yang T, Wang M, Debinskie W, Zhao W, Chan MD, Zhou X (2016). Identification of biomarkers for pseudo and true progression of GBM based on radiogenomics study. Oncotarget.

[218] Zinn PO, Majadan B, Sathyan P, Singh SK, Majumder S, Jolesz FA, Colen RR (2011). Radiogenomic Mapping of Edema/Cellular Invasion MRI-Phenotypes in Glioblastoma Multiforme. PLoS One.

[219] Jamshidi N, Diehn M, Bredel M, Kuo MD (2014). Illuminating Radiogenomic Characteristics of Glioblastoma Multiforme through Integration of MR Imaging, Messenger RNA Expression, and DNA Copy Number Variation. Radiology.

[220] Dai J, Shi J, Soodeen-Lalloo AK, Zhang P, Yang Y, Wu C, Jiang S, Jia X, Fei K, Jiang G (2016). Air bronchogram: A potential indicator of epidermal growth factor receptor mutation in pulmonary subsolid nodules. Lung Cancer.

[221] Kobayashi Y, Mitsudomi T, Sakao Y, Yatabe Y (2015). Genetic features of pulmonary adenocarcinoma presenting with ground-glass nodules: the differences between nodules with and without growth. Ann Oncol.

[222] Lee HJ, Kim YT, Kang CH, Zhao B, Tan Y, Schwartz LH, Persigehl T, Jeon YK, Chung DH (2013). Epidermal growth factor receptor mutation in lung adenocarcinomas: relationship with CT characteristics and histologic subtypes. Radiology.

[223] Shin DY, Na II, Kim CH, Park S, Baek H, Yang SH (2014). EGFR mutation and brain metastasis in pulmonary adenocarcinomas. J Thorac Oncol.

[224] Takano K, Kinoshita M, Takagaki M, Sakai M, Tateishi S, Achiha T, Hirayama R, Nishino K, Uchida J, Kumagai T, Okami J, Kawaguchi A, Hashimoto N (2016). Different spatial distributions of brain metastases from lung cancer by histological subtype and mutation status of epidermal growth factor receptor. Neuro Oncol.

[225] Sekine A, Kato T, Hagiwara E, Shinohara T, Komagata T, Iwasawa T, Satoh H, Tamura K, Kasamatsu T, Hayashihara K, Saito T, Takahashi H, Ogura T (2012). Metastatic brain tumors from non-small cell lung cancer with EGFR mutations: distinguishing influence of exon 19 deletion on radiographic features. Lung Cancer.

[226] Plodkowski AJ, Drilon A, Halpenny DF, O’Driscoll D, Blair D, Litvak AM, Zheng J, Moskowitz CS, Ginsberg MS (2015). From genotype to phenotype: Are there imaging characteristics associated with lung adenocarcinomas harboring RET and ROS1 rearrangements?. Lung Cancer.

[227] Park EA, Lee HJ, Kim YT, Kang CH, Kang KW, Jeon YK, Goo JM, Lee CH, Park CM (2009). EGFR gene copy number in adenocarcinoma of the lung by FISH analysis: investigation of significantly related factors on CT, FDG-PET, and histopathology. Lung Cancer.

[228] Halpenny DF, Riely GJ, Hayes S, Yu H, Zheng J, Moskowitz CS, Ginsberg MS (2014). Are there imaging characteristics associated with lung adenocarcinomas harboring ALK rearrangements?. Lung Cancer.

[229] Park J, Kobayashi Y, Urayama KY, Yamaura H, Yatabe Y, Hida T (2016). Imaging Characteristics of Driver Mutations in EGFR, KRAS, and ALK among Treatment-Naïve Patients with Advanced Lung Adenocarcinoma. PLoS One.

[230] Wang H, Schabath MB, Liu Y, Han Y, Li Q, Gillies RJ, Ye Z (2016). Clinical and CT characteristics of surgically resected lung adenocarcinomas harboring ALK rearrangements or EGFR mutations. Eur J Radiol.

[231] Yamamoto S, Korn RL, Oklu R, Migdal C, Gotway MB, Weiss GJ, Iafrate AJ, Kim DW, Kuo MD (2014). ALK molecular phenotype in non-small cell lung cancer: CT radiogenomic characterization. Radiology.

[232] Lanic H, Mareschal S, Mechken F, Picquenot J, Cornic M, Maingonnat C, Bertrand P, Clatot F, Bohers E, Stamatoullas A, Leprêtre S, Rainville V, Ruminy P (2012). Interim positron emission tomography scan associated with international prognostic index and germinal center B cell-like signature as prognostic index in diffuse large B-cell lymphoma. Leuk Lymphoma.

[233] Tateishi U, Hasegawa T, Nojima T, Takegami T, Arai Y (2006). MRI features of extraskeletal myxoid chondrosarcoma. Skeletal Radiol.

[234] Brisson M, Kashima T, Delaney D, Tirabosco R, Clarke A, Cro S, Flanagan AM, O’Donnell P (2013). MRI characteristics of lipoma and atypical lipomatous tumor/well- differentiated liposarcoma: Retrospective comparison with histology and MDM2 gene amplification. Skeletal Radiol.

[235] Bordia R, Zhong H, Lee J, Weiss S, Won S (2017). Melanoma brain metastases : correlation of imaging features with genomic markers and patient survival. J Neurooncol.

[236] Mai EK, Hielscher T, Kloth JK, Merz M, Shah S, Hillengass M, Wagner B, Hose D, Raab MS, Jauch A, Delorme S, Goldschmidt H, Weber M (2016). Association between magnetic resonance imaging patterns and baseline disease features in multiple myeloma : analyzing surrogates of tumour mass and biology. European Radiology.

[237] Vargas HA, Miccò M, Hong SI, Goldman DA, Dao F, Weigelt B, Soslow RA, Hricak H, Levine DA, Sala E (2015). Association between morphologic CT imaging traits and prognostically relevant gene signatures in women with high-grade serous ovarian cancer: a hypothesis-generating study. Radiology.

[238] Liu Y, Lu MY, Chang H, Lu C, Lin DT, Jou S, Yang Y, Lee YL, Huang S, Jeng YM, Lee H, Miser JS, Lin K (2016). Diagnostic FDG and FDOPA positron emission tomography scans distinguish the genomic type and treatment outcome of neuroblastoma. Oncotarget.

[239] Perreault S, Ramaswamy V, Achrol AS, Chao K, Liu TT, Shih D, Remke M, Schubert S, Bouffet E, Fisher PG, Partap S, Vogel H, Taylor MD (2014). MRI surrogates for molecular subgroups of medulloblastoma. AJNR Am J Neuroradiol.

[240] Stoyanova R, Pollack A, Takhar M, Lynne C, Parra N, Lam LL, Alshalalfa M, Buerki C, Castillo R, Jorda M, Ashab HA, Kryvenko ON, Punnen S (2016). Association of multiparametric MRI quantitative imaging features with prostate cancer gene expression in MRI-targeted prostate biopsies. Oncotarget.

[241] Nagarajah J, Ho AL, Tuttle RM, Weber WA, Grewal RK (2015). Correlation of BRAFV600E Mutation and Glucose Metabolism in Thyroid Cancer Patients: An (1)(8)F-FDG PET Study. J Nucl Med.

