# Supplementary material for: Non-invasive tumor genotyping using radiogenomic biomarkers, a systematic review and oncology-wide pathway analysis
Source: Oncotarget. 2018 Apr 13;9(28):20134–55. doi: 10.18632/oncotarget.24893 (PMC5929452; doi:10.18632/oncotarget.24893)
Supplement: Supplementary file 1 [file oncotarget-09-20134-s001.pdf]

Non-invasive tumor genotyping using radiogenomic biomarkers, a systematic review and oncology-wide pathway analysis

SUPPLEMENTARY MATERIALS

Supplementary Table 1: Search strategy and inclusion flowchart. See Supplementary_Table_1

Supplementary Table 2: Database of radiogenomic associations. See Supplementary_Table_2

Supplementary Table 3: Study characteristics and quality assessment of included studies. See Supplementary_Table_3

Supplementary Table 4: Radiogenomic associations for *MGMT*-status in diffuse glioma. See Supplementary_Table_4

Supplementary Table 5: Radiogenomic associations for *EGFR*-status in diffuse glioma. See Supplementary_Table_5

Supplementary Table 6: Radiogenomic associations for *KRAS*-status in NSCLC. See Supplementary_Table_6

Supplementary Table 7: Significant radiogenomic relations repeatedly found in different types of cancer. See Supplementary_Table_7

Supplementary Table 8: Overview of radiogenomics for predicting 1p/19q codeletion in glioma grade II-III, *p*-values for associations. See Supplementary_Table_8

Supplementary Table 9: Studies using multiparametric modelling for radiogenomics in GBM. See Supplementary_Table_9

Supplementary Table 10: Overview of radiogenomics for EGFR mutations and ALK rearrangements in non-small cell lung cancer, *p*-values for associations. See Supplementary_Table_10
